# Linear coding of complex sound spectra by discharge rate in neurons of the medial nucleus of the trapezoid body (MNTB) and its inputs

**DOI:** 10.3389/fncir.2014.00144

**Published:** 2014-12-16

**Authors:** Kanthaiah Koka, Daniel J. Tollin

**Affiliations:** Department of Physiology and Biophysics, University of Colorado School of MedicineAurora, CO, USA

**Keywords:** calyx of held, medial nucleus of the trapezoid body, lateral superior olive, spectrotemporal receptive field, sound localization, temporal processing

## Abstract

The interaural level difference (ILD) cue to sound location is first encoded in the lateral superior olive (LSO). ILD sensitivity results because the LSO receives excitatory input from the ipsilateral cochlear nucleus and inhibitory input indirectly from the contralateral cochlear nucleus via glycinergic neurons of the ipsilateral medial nucleus of the trapezoid body (MNTB). It is hypothesized that in order for LSO neurons to encode ILDs, the sound spectra at both ears must be accurately encoded via spike rate by their afferents. This spectral-coding hypothesis has not been directly tested in MNTB, likely because MNTB neurons have been mostly described and studied recently in regards to their abilities to encode temporal aspects of sounds, not spectral. Here, we test the hypothesis that MNTB neurons and their inputs from the cochlear nucleus and auditory nerve code sound spectra via discharge rate. The Random Spectral Shape (RSS) method was used to estimate how the levels of 100-ms duration spectrally stationary stimuli were weighted, both linearly and non-linearly, across a wide band of frequencies. In general, MNTB neurons, and their globular bushy cell inputs, were found to be well-modeled by a linear weighting of spectra demonstrating that the pathways through the MNTB can accurately encode sound spectra including those resulting from the acoustical cues to sound location provided by head-related directional transfer functions (DTFs). Together with the anatomical and biophysical specializations for timing in the MNTB-LSO complex, these mechanisms may allow ILDs to be computed for complex stimuli with rapid spectrotemporally-modulated envelopes such as speech and animal vocalizations and moving sound sources.

## Introduction

The interaural level difference (ILD) cue to sound location requires neural encoding of the shapes and magnitudes of sound spectra. ILDs result from frequency- and direction-dependent modifications of sound by the head and pinnae and are defined as the difference in spectra of the signals at the two ears (Tollin and Koka, 2009a,b). In the mammalian brainstem, the superior olivary complex contains a circuit comprising the ipsilateral medial nucleus of the trapezoid body (MNTB) and the lateral superior olive (LSO) that is essential for ILD encoding (Tollin, 2003). LSO neurons receive excitatory input from spherical bushy cells (SBCs) of the ipsilateral cochlear nucleus (CN) and inhibitory input from the contralateral ear via the MNTB; the MNTB receives excitatory input from globular bushy cells (GBCs) of the contralateral CN. SBCs and GBCs receive excitatory inputs from the auditory nerve. These inputs confer upon single LSO neurons the ability to compute a neural correlate of ILDs (Boudreau and Tsuchitani, 1968).

Although there is consensus that the LSO initially encodes ILDs and that the inhibitory input to the LSO from the MNTB is essential, the mechanisms are still not well understood. The accurate and precise encoding of ILD observed in LSO neurons (e.g., Tollin et al., 2008) would seem to imply that the neurons comprising the ascending inputs to LSO must be accurately encoding sound spectra at the two ears. However, this hypothesis has not been explicitly tested. One reason for this may be that the MNTB and bushy cells are mostly described, and thus studied, in regards to their exquisite abilities to encode *temporal* aspects of sounds (Wu and Kelly, 1993; Taschenberger and Von Gersdorff, 2000; Futai et al., 2001; Joshi et al., 2004; Lorteije et al., 2009) but not spectral. The exquisite temporal processing capabilities of MNTB result from several specializations. First, the input from GBCs onto MNTB neurons forms the largest, most secure synapses in the CNS, the calyx of Held (Jean-Baptiste and Morest, 1975; McLaughlin et al., 2008). Each MNTB neuron receives only a single calyx, which can envelop up to half the soma surface, and large pre-synaptic terminals that produce large post-synaptic currents (Banks and Smith, 1992; Smith et al., 1998), facts that have made this synapse a model for synaptic transmission (Forsythe, 1994; Borst et al., 1995; Schneggenburger and Forsythe, 2006). MNTB neurons have short membrane time constants, receptors with fast kinetics, and specialized ion channels that together with specializations in the calyx result in large, rapid EPSPs that excite MNTB neurons with nearly invariant synaptic delays (Wu and Kelly, 1991; Banks and Smith, 1992; von Gersdorff and Borst, 2002; Trussell, 2002; although see Tolnai et al., 2009) making them indeed well suited to preserve temporal information that is important for the encoding of the binaural cues to sound location (Joris and Yin, 1998; Tollin and Yin, 2005).

Despite these extraordinary specializations for temporal fidelity, we hypothesize that MNTB neurons must also accurately code the shapes of the sound spectra at the ears over short time intervals in order to account for the abilities of LSO neurons to encode the frequency-dependent acoustic ILDs (Tollin and Yin, 2002a,b; Tollin et al., 2008; Tsai et al., 2010) and for animals such as cats to use these ILD cues to accurately and precisely localize high-frequency sound sources (Tollin et al., 2005, 2013; Moore et al., 2008; Gai et al., 2013; Ruhland et al., 2013). Here, a systems identification method, the Random Spectral Shape (RSS) technique (Yu and Young, 2000), was used to test the hypothesis that MNTB neurons and their inputs, the GBCs and auditory nerve fibers, encode stationary sound spectra linearly via their discharge rate. The RSS technique estimates the spectral weighting function that describes how spectra are linearly and non-linearly weighted to produce a discharge rate. Both GBC and MNTB neurons were well modeled by a linear weighting of sound spectra, consistent with previous reports in auditory nerve and other CN neurons (Yu and Young, 2000, 2013; Young and Calhoun, 2005). Together with the anatomical and biophysical specializations for timing in the neural circuits comprising the GBC, MNTB, and LSO, the mechanisms that produce accurate linear coding of spectral levels in these neurons may allow ILD cues to be coded for complex biologically-relevant stimuli with rapid spectrotemporally-modulated envelopes such as speech and animal vocalizations and moving sound sources.

## Materials and methods

### Animals, apparatus, and experimental procedures

All surgical and experimental procedures complied with the guidelines of the University of Colorado Anschutz Medical Campus Animal Care and Use Committee and the National Institutes of Health. Methods are based on those described in Tollin et al. (2008) and Tsai et al. (2010). Adult cats with clean external ears were initially anesthetized with ketamine hydrochloride (20 mg/kg) along with acepromazine (0.1 mg/kg). Atropine sulfate (0.05 mg/kg) was also given to reduce mucous secretions, and a tracheal cannula was inserted. Supplemental doses of sodium pentobarbital (3–5 mg/kg) were administered intravenously into the femoral vein as needed to maintain areflexia. Heart rate was continuously monitored as was core body temperature (with a rectal probe), the latter maintained with a heating pad at 37°C (Model TC 100, CWE, Inc., Ardmore, PA). Blood-oxygen levels, respiratory rate, and end-tidal CO_2_ were measured continuously via a capnograph (Surgivet V90040, Waukesha, WI) and mean arterial blood pressure (femoral artery) was monitored with a pressure transducer (Harvard Apparatus research blood pressure transducer, Holliston, MA). Both pinnae were cut transversely, removed, and tight-fitting custom built hollow earpieces were fitted tightly into the external auditory meati. Polyethylene tubing (Intramedic, PE-90, 30 cm, 0.9 mm ID) was glued into a small hole made in each bulla to maintain normal middle ear pressure.

The trapezoid body and the MNTB was approached ventrally by drilling small holes into the basioccipital bone. Parylene-coated tungsten microelectrodes (1–2 MΩ, Microprobe, Clarksburg, MD) were advanced ventromedially to dorsolaterally at an angle of 26–30° into the brainstem by a microdrive (Kopf Model 662, Tujunga, CA) affixed to a micromanipulator that could be remotely advanced from outside the double-walled sound-attenuating chamber (Industrial Acoustics, Bronx, NY). Electrical activity was amplified (ISO-80, WPI, Sarasota, FL) and filtered (300–3000 Hz; Stanford Research Systems SRS 560, Sunnyvale, CA). Unit responses were discriminated with a BAK amplitude-time window discriminator (Model DDIS-1, Mount Airy, MD) and spike times were stored at a precision of 1 μs via a Tucker-Davis Technologies (TDT, Alachua, FL) RV8.

#### Stimuli: general

All stimuli were generated digitally at 24-bit resolution and converted to analog at a nominal rate of 100 kHz by a TDT RX-6. Overall stimulus level to each ear was independently controlled in 1 dB steps using a pair of TDT PA-5s. The conditioned output of the D/A converter was sent to an acoustic assembly (one for each ear) comprising a TDT EC1 electrostatic speaker, a calibrated probe-tube microphone (Bruel and Kjaer Type 4182, Norcross, GA), and a hollow earpiece that was fit tightly into the cut end of the auditory meatus and sealed with petroleum jelly. The hollow earpiece accommodated the small probe-tube microphone by which the sound delivery system to each ear was calibrated for tones between 50 Hz and 40 kHz in 50–100 Hz steps. The calibration data was used to compute 256 tap Finite Impulse Response digital filters that equalized the responses of the acoustical system and typically yielded flat frequency responses within ±2 dB for frequencies less than 35 kHz (Koka et al., 2010).

Tone bursts of varying frequency were used as search stimuli. Once a single unit was isolated, the characteristic frequency (CF), spontaneous activity, and threshold were measured using an automated threshold tracking routine or by measuring a frequency-intensity response area (±2 octaves around the CF with 1/8 octave frequency increments and ~0–80 dB SPL in 5 dB increments). The sharpness of the tuning curves was measured as the Q_10_ (CF/bandwidth at 10 dB above threshold). Rate-level functions were measured by presenting 200 repetitions of a 50-ms tone pip at CF (5-ms rise-fall times) every 100 ms from which the resulting PST histograms (PSTHs) were examined on-line. For some neurons, the sensitivity to ILDs was examined by holding the sound level presented to the contralateral, excitatory ear constant at ~20 dB above the contralateral-ear only threshold level and varying the stimulus level to the ipsilateral ear ±25 dB about the contralateral ear sound level (i.e., ILDs varied between ±25 dB). Discharge rate vs. sound level functions were also measured for 100-ms duration flat-spectrum broadband noise by presenting 20 repetitions of the noise at each stimulus level tested.

#### MNTB and GBC neuron classification

When recording extracellularly with metal electrodes, care must be taken in positively categorizing neurons as MNTB due to the presence of the large numbers of fibers of the trapezoid body passing directly through the MNTB; many of these fibers respond to sound stimuli similar to MNTB neurons (Smith et al., 1991, 1993). Here, the criteria of Smith et al. (1998) were used to classify MNTB principal cells based on their extracellular responses. Neurons were classified as MNTB based on three properties: (1) responses only to stimuli presented to the contralateral ear (Guinan et al., 1972a,b; Smith et al., 1998); (2) the presence of a prepotential in the action potential waveform (Guinan and Li, 1990); and (3) a primary-like (PL) or a primary-like with notch (PLN) PSTH to short tone burst stimuli (Smith et al., 1998). GBC-like responses were obtained from fiber recordings in the trapezoid body. GBCs fibers were also classified according to Smith et al. (1991, 1998) by monaural-only responses, PLN or onset-L (OnL; Rhode, 2008) PSTHs to short tone stimuli, the lack of a pre-potential in the extracellular waveform, and a more ventral recording depth than MNTB. This is a conservative characterization of GBCs from electrophysiological responses because some morphologically-identified GBCs can have primary-like PSTHs to tones at some stimulus levels (Rhode, 2008).

#### Histology

In many experiments, electrolytic DC lesions (5 μA × 10 s) were made to differentiate electrode tracks, mark locations of interest, and assist in estimating tissue shrinkage after histological processing. At the conclusion of each experiment, the brain was fixed in formalin or 4% buffered paraformaldehyde by immersion or perfusion through the heart. The fixed tissue was cut into 50-μm frozen sections and stained with cresyl violet so that electrode tracks and lesions made during the recordings could be seen.

#### RSS method and spectral weight function model

**Figure 1:**
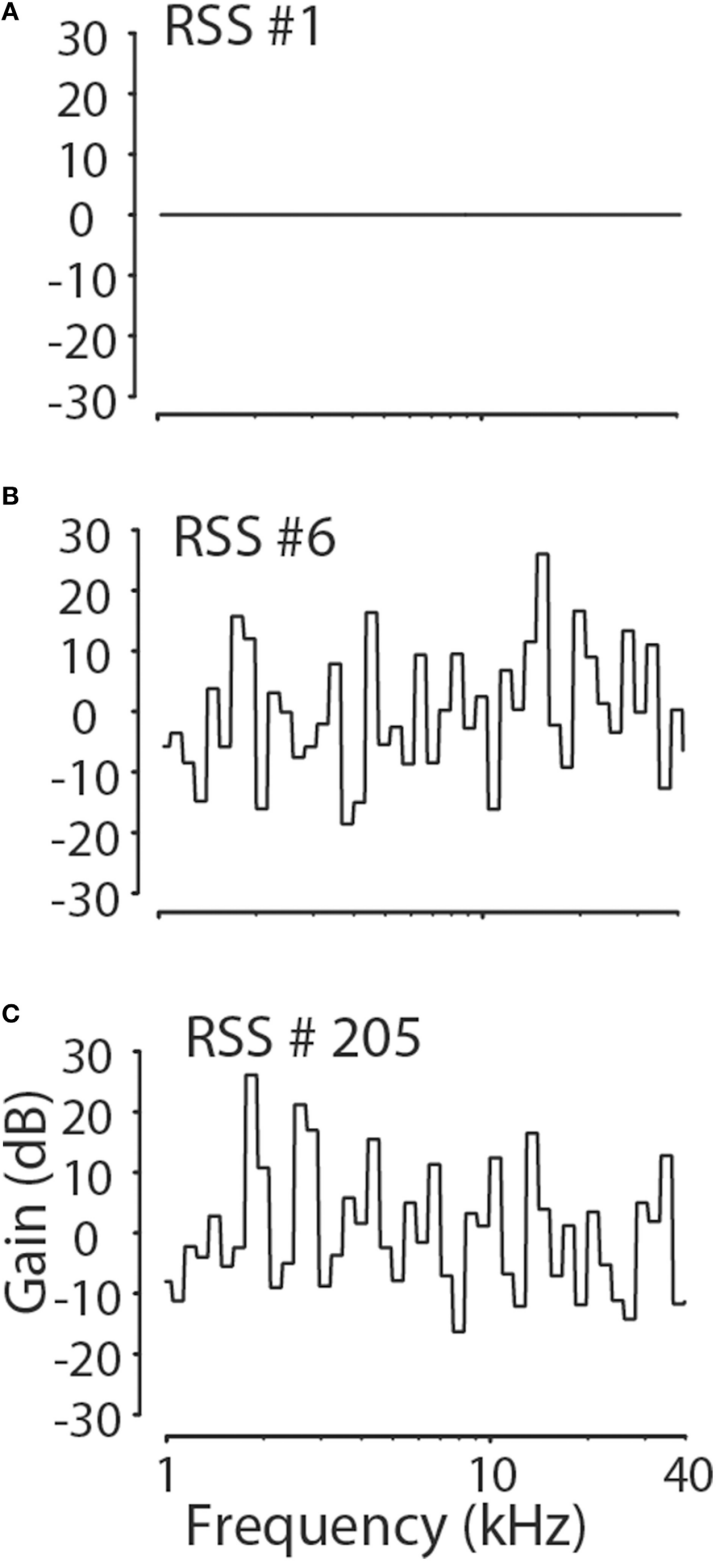
**Examples of three of 264 RSS stimulus spectra**. The RSS stimuli had spectral contrasts chosen from a normal distribution with a standard deviation of 10 dB. Four of 264 stimuli had flat spectra **(A)**, while the remaining stimuli had spectra resembling those in **(B,C)**.

The RSS technique is a systems identification method (Yu and Young, 2000, 2013; Young and Calhoun, 2005; Bandyopadhyay et al., 2007; Reiss et al., 2007) to determine how neurons linearly and non-linearly weight sound spectra to increase or decrease their discharge rate. For this paper, 264 different pseudorandom RSS noises were created, each consisting of the sum of 512 random-phase tones spaced logarithmatically in frequency at 1/64-octave spacing and covering the range from 0.17 to 40 kHz. The random phase of the tones eliminates the formation of an onset transient after the summation of the tones. The tones were grouped into 64 frequency bins each containing 8 tones, so that each bin spanned 1/8 octave. Relative to the mean overall spectral level of each stimulus (i.e., a flat-spectrum broadband noise where all bins are set to a gain of 0 dB), the amplitude of the tones in each of the 64 bins, *S*(*f*) in dB, were chosen randomly from a normal distribution that had a mean and standard deviation of 0 and 10 dB, respectively, so that all 8 components in a single bin have the same amplitude. Of the 264 RSS stimuli 4 had a flat spectrum, *S*(*f*) = 0 dB for all *f*, while the remaining 260 stimuli had spectral patterns as just described. Figure 1 shows the amplitude spectra in terms of the gain in dB re: the reference level for three of the stimuli among 264 stimuli, including one with a flat spectrum.

Across the 264 stimuli, the amplitudes in each frequency bin were specifically constructed to be uncorrelated with the amplitudes in all other bins. This constraint allows the use of linear least squares techniques (Press et al., 1986) to compute the spectral weights from the rate responses to the ensemble of RSS stimuli. Moreover, the zero mean and uncorrelated spectral levels in the different frequency bins allows the computation of both first order and second order weighting functions separately. In order for this to occur, as described by Reiss et al. (2007), the ensemble of RSS stimuli were ordered into successive plus-minus pairs such that the spectral levels of the first stimulus of the pair *S*_i_(*f*) were simply inverted in the second stimulus *S*_*i*+1_(*f*) [i.e., *S*_*i*+1_(*f*) = −*S*_*i*_(*f*)]. These plus-minus pairs of stimulus were used to separate the estimation of the even and the odd order terms in the model presented below. The full description and validation of this technique can be found in Reiss et al. (2007).

The ensemble of 264 RSS stimuli was presented at 4–10 overall levels spanning ~20 dB below to ~40 dB above the threshold level for the flat spectrum noise alone. The threshold was estimated to within ±2.5 dB from the rate-level function for one of the flat-spectrum RSS stimuli. In all cases, the RSS stimuli were 100 ms in duration and were gated on and off with 10-ms linear ramps.

The weight function model is based on the following equation for average discharge rate *r* of a neuron computed over the 100-ms duration of the stimulus:

(1a) $$r=R_{0}+\sum_{j\;=\;1}w_{j}S(f_{i})+\sum_{j\;=\;1}\sum_{k\;=\;j}w_{jk}S(f_{i})S(f_{k})$$

which can be re-written in matrix form in the following way:

(1b) $$r=R_{0}+{\text{w}}^{\text{T}}\text{s}+{\text{s}}^{\text{T}}\text{Ms}$$

where s is a vector containing the dB values of each stimulus at different frequencies [i.e., *S*(*f*_*i*_)], w is a vector containing the first order, or linear, weights (i.e., *w*_*j*_) of the neuron [in units of spikes/(s·dB)], M is a matrix of second order, or non-linear, weights (i.e., *w*_*jk*_) of the neuron [units of spikes/(s·dB^2^)] and T indicates transposition. The matrix of second order weights measures the contribution to the response of the neuron to quadratic terms like the energy-squared at a particular frequency [e.g., *w*_*jj*_ for *S*^2^(*f*_*j*_)] or the product of the energy at two different frequencies. Finally *R*_0_ is a constant, which is the rate response to the flat spectrum stimulus with all frequency bins set to 0 dB level. For all the calculations, dB levels were not always corrected for the speaker calibration; however, because the headphone calibrations are locally flat (re: the spectral receptive field of a given neuron), correcting for the calibration had negligible effects on the data (see also Young and Calhoun, 2005).

For each neuron the model parameters for Equation (1) were estimated using the discharge rates in responses to single presentations of each of the 264 RSS stimuli, which thus results in 264 equations as expressed by Equation (1). The variable *R*_0_ was estimated directly as part of Equation (1b). The first- and second-order model weights, w and M Equation (1b), were estimated by the method of normal equations (e.g., Press et al., 1986) by minimizing the chi-square error:

(2) $$\chi^{2}(w,M)=\sum_{j}\frac{{\left[r_{j}-\overset{\wedge }{r_{j}}\left(s_{j},w,M\right)\right]}^{2}}{\sigma_{j}^{2}}$$

Here *r*_*j*_ are the empirical rates measured in the experiment and *r*_*j*_(s_*j*_,w,M) are the rates predicted by the model in Equation (1) for the stimulus s_*j*_ and the first and second-order weights w and M, respectively, and σ^2^_*j*_ is the variance of the rate response *r*_*j*_. We did not attempt to estimate directly σ^2^_*j*_ (the variance of the rates computed over multiple repetitions of the same stimulus) in all neurons due to the limited recording time for each neuron. Instead, we assumed that the response counts (i.e., *r*_*j*_/*T*, where *T* = 100 ms, the duration of the stimuli used here) were Poisson distributed such that the variance of the count was equal to the mean count. Also, to avoid having the denominator in Equation (2) go to zero, σ^2^_*j*_ was not allowed to have a value <0.1. Using Equations (1) and (2), the model weights were computed using the weighted least squares technique (Press et al., 1986) in MATLAB (v7.1, The Mathworks, Inc., Natick, MA), where the diagonal of the weight matrix is equal to σ^2^_*j*_.

As one check of the applicability of the Poisson variance assumption, in 8 neurons the response variance σ^2^_*j*_ was estimated from a power-law fit of the response variance vs. the mean response computed from the flat-spectrum stimulus rate-level function where multiple presentations of the same stimuli were presented (see Tollin et al., 2008). From the power-law fitted function, given an arbitrary discharge rate, the corresponding rate variance could be accurately predicted. Using this empirically-determined response variance instead of the Poisson variance assumption did not materially change the weight functions (e.g., see **Figures 3A1,B1**) or the predicted responses to arbitrary stimuli not used in the fitting of the model parameters (see below).

Computational estimation of the first- and second-order weights was simplified due to the design of the plus-minus RSS stimulus pairs as mentioned earlier. Following from Reiss et al. (2007), let *r*^+^ and *r*^−^ be the rates in response to a plus-minus stimulus pair *s*^+^ and *s*^−^ (i.e., where *s*^+^ = − *s*^−^). From Equation (1):

(3) $$\frac{r^{+}+r^{-}}{2}=R_{0}+{\text{s}}^{+\text{T}}{\text{Ms}}^{+}\text{ and }\frac{r^{+}-r^{-}}{2}={\text{s}}^{+\text{T}}\text{w}$$

Here, the estimation of the second-order weight matrix M is based on (*r*^+^ + *r*^−^)/2 and the first order weight vector w based on (*r*^+^ − *r*^−^)/2. As before, *R*_0_ is estimated either from the responses to the flat spectrum RSS stimuli or as part of the parameters in Equation (2). For each neuron and overall sound level we used that *R*_0_ which maximized the fraction of explained variance (*fv*, see next section). We adopted this procedure because the *R*_0_ measured from only four presentations of the flat-spectrum stimuli did not always maximize *fv*; more than four presentations of the flat spectrum stimuli apparently need to be measured to get a more accurate estimate of *R*_0_. Because the estimates of different orders of weights are not necessarily orthogonal (see Reiss et al., 2007), if the neuron were to actually be influenced by third (or higher) order weights, this would appear as an error in the estimation of the lower-order weights. Estimating the first and second order weights separately in the way described above reduces this error by keeping the error from un-estimated odd-order components from affecting even-order estimates and vice versa.

The standard deviations (SDs) of the spectral weights were estimated using standard statistical bootstrapping techniques (Efron and Tibshirani, 1993). This was done because neural responses to multiple repetitions of stimuli were not collected in all neurons. The SDs of the weights were computed in the following way. A set of 200 RSS stimulus/response pairs were chosen at random with replacement from the 200 RSS stimuli estimation set (see next section) and the spectral weight functions computed using Equation (1b). This process of selecting stimulus/response pairs with replacement and computing the spectral weights was repeated at least 200 times. From the resulting 200 spectral weight functions the mean and SD of each the weights were calculated.

#### Prediction of responses to arbitrary stimuli: quality of the model fit

A rigorous test of the spectral weight model Equation (1a) was the ability to predict the rate responses to arbitrary stimuli that were not used in the fitting process. To this end, the 264 RSS stimuli were divided in to two groups: (1) an estimation set of the rate responses to 200 RSS stimuli [1–100 from positive-spectra half and 1–100 from negative-spectra half, see Equation (3)] and (2) a prediction set consisting of the responses to the remaining 64 RSS stimuli (101–132 from positive half and 101–132 from negative half). The set of 200 RSS stimuli was used to estimate the spectral weights using Equation (1b). These weights were then used to predict the responses to the 64 stimuli also using Equation (1b). The quality of the model was quantified by an adaptation of the fraction of unexplained variance (Hays, 1988) which has been defined by Young and Calhoun (2005) as:

(4) $$fv=1-\frac{\sum {\left(r_{j}-{\hat{r}}_{j}\right)}^{2}}{\sum {\left(r_{j}-\bar{r}\right)}^{2}}$$

Here, for the *i*th RSS stimulus, *r*_*j*_ the empirical rate, $\hat{r}$_*j*_ is the rate predicted by the model, and *r* is the mean rate computed over all RSS stimuli. *fv* values vary from a maximum of 1 (perfect fit) and decrease with poorer predictions; *fv* can take values <0 when the fit is particularly poor. The *fv* was also used to assist in the determination of the numbers of first- and second-order model parameters for the spectral weight functions. The numbers of spectral weights were systematically added to the model beginning with the weight at BF, along with the corresponding number of second-order weights. More weights were added until the *fv* for prediction was maximized. The frequency range of the weights was typically within one octave below and one-half octave above BF. Choosing weights in this way helps to avoid over fitting the model. The correlation coefficients were also quantified for the predictions along with the *fv* values and compared.

#### Rationale for using the random spectral shape technique

For neurons in the auditory system with complex receptive fields, researchers often characterize the so-called spectrotemporal receptive field (STRF; Aertsen et al., 1981; Eggermont, 1993; Kowalski et al., 1996; deCharms et al., 1998; Theunissen et al., 2000; Schnupp et al., 2001; Escabi and Schreiner, 2002). The STRF estimates the average power spectrum of the stimulus as a function of time (e.g., the spectrogram) preceding an action potential elicited from a neuron. Here, it is assumed that the STRFs for MNTB neurons are spectrally and temporally separable, which is a good assumption for neurons peripheral to the inferior colliculus (Qiu et al., 2003; Lewis and van Dijk, 2004; Lesica and Grothe, 2008; Versnel et al., 2009). When any separable STRF is averaged over time, the temporal component in any particular frequency bin reduces to a constant. The resultant STRF summation, then, gives rise to precisely the RSS weight function as given in Equation (1) (Young et al., 2005). Moreover, for stimulus sets for which the spectra are stationary (i.e., fixed throughout the stimulus duration), such as the RSS stimuli used here, any residual spectral-temporal interactions may be minimized (Young et al., 2005). While temporal interactions probably do play an important role in establishing the ultimate responsiveness of MNTB neurons (see Kopp-Scheinpflug et al., 2008), we wanted to test here the specific hypothesis that the neurons of the MNTB accurately encode stationary spectral characteristics of the stimuli via discharge rate. Toward this specific goal, the RSS technique (Yu and Young, 2000) represents an efficient method.

#### Responses to acoustic directional transfer function-filtered broadband noise stimuli

As an additional test of the weight function model, a behaviorally-relevant set of acoustical stimuli was used. Here, the first- and second-order weights of the estimated spectral weight models for each neuron tested were used to predict the responses to 100-ms duration flat-spectrum broadband noise filtered by directional transfer functions (DTFs) from 325 to 627 locations in the front and rear hemispheres. Acoustic DTFs contain the sound source location dependent features of the head related transfer functions (HRTF) (Koka et al., 2011). The acoustic DTFs were measured in each animal in this paper immediately prior to the physiological studies. The methods for measuring the DTFs and the associated acoustical cues to location computed from them are described in detail in Tollin and Koka (2009a,b). Spatial plotting of the DTF-stimuli and the neural responses to them was done using Aitov projections. These spatial plots are shown in this paper for just the frontal hemisphere from elevations −45° to +90° and azimuths −90° (contralateral to MNTB being studied) to +90°. This area consists of 325 DTF filtered stimuli. The fraction of variance *fv* was quantified for the predicted rate responses for DTF stimuli. Two-dimensional smoothing was done on empirical responses along with responses predicted from either first order alone or first order and second order together for plotting purposes only. The spatial correlation coefficients were calculated and compared along with the *fv* values. The spatial correlation coefficients explain how well the model can predict the general two-dimensional shape of the neural spatial receptive fields.

## Results

**Figure 2:**
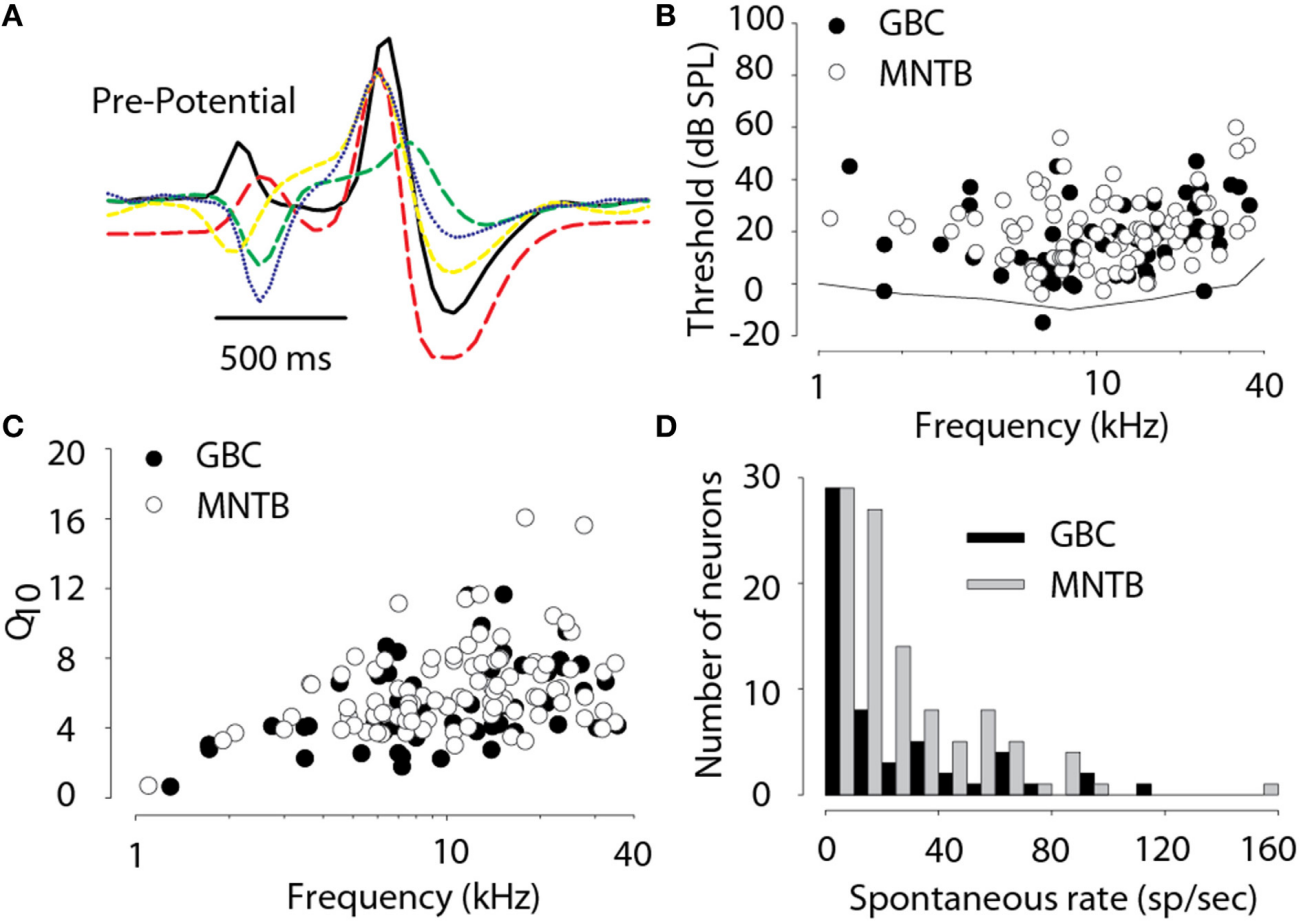
**(A)** Examples of extracellular voltage waveforms illustrating the pre-potential component in 5 MNTB neurons. **(B)** Threshold SPL for tones as a function of the characteristic frequency of each neuron. Behavioral audiogram for cat from Heffner and Heffner (1985) (solid line). **(C)** Frequency tuning bandwidth, Q_10_ (characteristic frequency divided by the bandwidth 10 dB above threshold), as a function of characteristic frequency. **(D)** Histogram of spontaneous activity. Data in **(B–D)** are based on 103 MNTB and 51 GBC neurons.

Results are based on recordings of 103 MNTB and 51 GBC neurons collected from 22 subjects. All 103 MNTB neurons exhibited a complex “pre-potential” that preceded the action potential by ~0.5–0.7 ms and all responded only to stimuli presented to the contralateral ear. Figure 2A shows examples of the complex waveforms of the extracellularly-recorded action potentials from five neurons. The diversity in waveform shapes is consistent with other reported recordings in cat MNTB (e.g., Guinan and Li, 1990; Joris and Yin, 1998; Smith et al., 1998). Electrode tracks were able to be reconstructed from the histology in 8 cats, allowing the verification of 44/103 neurons in the MNTB. The location of neurons as a function of their CF was in agreement with prior studies (Guinan et al., 1972a; Sommer et al., 1993; Smith et al., 1998; Tollin and Yin, 2005) with high-CF neurons located medioventral and lower-CF neurons located dorsolateral in the nucleus. Histology was either not available or the electrode tracks could not be reconstructed to localize the remaining neurons. These remaining neurons are included here because they all had properties consistent with MNTB neurons in the cat: complex “pre-potential” wave shape (e.g., Figure 2A), responsive to contralateral ear stimulation only, gave PL or PLN response type, and where two or more MNTB neurons were recorded in the same electrode penetration there was systematically increasing progression of CF with electrode depth. Thirty eight MNTB neurons were studied extensively with 8 bin/oct RSS stimuli. Finally, 14 of these 38 neurons were studied with virtual acoustic space stimuli consisting of broadband noise appropriately filtered by a set of DTFs. Responses from 51 putative GBC fibers were recorded, 21 of which were studied with RSS stimuli.

### Basic acoustical response properties

Several basic acoustic response properties were measured for all 103 MNTB neurons and 51 GBC fibers. Figure 2B shows the threshold SPL as a function of CFs for all neurons and also the cat audiogram (Heffner and Heffner, 1985). The CFs ranged from 0.32 to 35 kHz (mean *CF* = 12.4 ± 8 kHz, median = 10.6 kHz) and 1.3–35.5 kHz (12.7 ± 8.44 kHz; 10.5 kHz) and thresholds ranged from -4 to 60 dB SPL (mean threshold = 20.5 ± 12.8, median = 20 dB) and −15 to 47 dB SPL (16.5 ± 14.0, 15.0) for MNTB and GBC, respectively. In MNTB the Q_10_ (Figure 2C) ranged from 0.5 to 16 and was highly dependent on CF with low Q_10_ of ~1–2 for CFs of <1 kHz increasing to ~8 for high CFs. The Q_10_ (Figure 2C) in GBCs ranged similarly across CF from 0.64 to 11.6. MNTB neurons exhibited a high degree of spontaneous activity (Figure 2D) ranging from 0 to 150 spikes/s (mean spontaneous activity = 25.5 ± 25.9 spikes/s; median = 17.5 spikes/s) while GBC fibers ranged from 0 to 40 spikes/s (23.6 ± 25.1; 13.0). In 20/20 (100%) MNTB neurons tested (CFs spanning 1.9–35 kHz) exhibited no sensitivity to ILDs (i.e., the responses were not affected by stimuli at the ipsilateral ear). Finally, the spontaneous activity and thresholds were not significantly different, as assessed by an independent-samples *t*-test [*t*_(152)_ < 1.8, *p* > 0.05 for both tests] between the GBC and MNTB neurons consistent with GBC providing MNTB with its afferent excitatory input via the calyx of Held; however MNTB neurons tended to have significantly, albeit slightly, narrower frequency selectivity than GBCs as assessed from Q_10_ (Figure 2C) [*t*_(152)_ = 2.0, *p* = 0.03].

### General properties of the spectral weight functions

**Figure 3:**
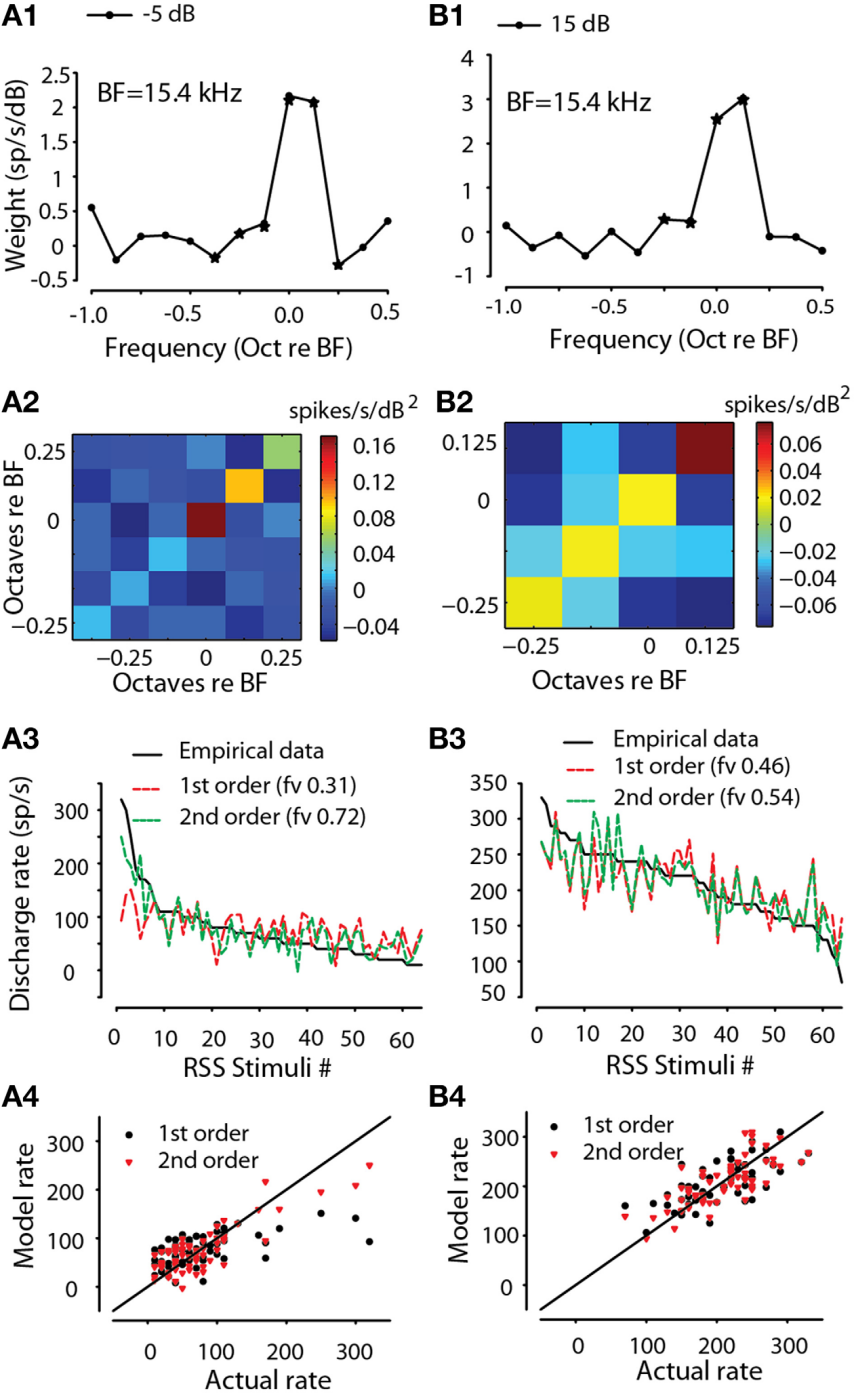
**Encoding of sound spectra by MNTB neuron via RSS method**. First order (linear) spectral weight functions for one neuron (*CF* = 15.4 kHz) at two levels re: threshold (**A1** −5 dB, **B1** 15 dB). Second-order weights for the same two levels **(A2,B2)**. Empirical discharge rates plotted rank-ordered vs. RSS stimuli (black line) along with the predicted rates using the estimated first-order (red line) and full-order (green line) spectral weight models for the same two levels **(A3,B3)**. The fraction of explained variance, *fv*, is indicated for each model. Scatter plot of modeled rate vs. empirical rate for first-order (black) and full-order (red) models for the same two levels **(A4,B4)**. Solid line is line of equality.

For all MNTB and GBC neurons, the first order weight functions, e.g., Equation (1a) showed an excitatory area (positive weights) near the neuron CF. The frequency bin corresponding to the largest spectral weight will be called the best frequency (BF). Figures 3A1,B1 (black symbols) show the first order weights for one MNTB neuron (*BF* = 15.4 kHz) taken at −5 and 15 dB sound levels above the flat-spectrum noise threshold, respectively. At both sound levels, the peak weight was at BF with smaller weights at adjacent higher and lower frequencies. Standard deviations of the weights (not shown) were estimated using statistical resampling techniques (Efron and Tibshirani 86). Weights ±1 SD away from 0 were considered significant and were subsequently used for predictions (see below); significant weights are indicated by a star symbol in Figures 3A1,B1. The second order weights (Figures 3A2,B2) were positive (red) at the BF and along the diagonal (i.e., same frequency bins on the x and y axes) but negative (blue) at the adjacent off diagonal frequencies. In general, the second order weights were about an order of magnitude smaller than the first order weights.

### Testing the validity of the spectral weight function model

The weight functions (Equation 1 and Figures 3A1,B1) are designed to quantify the transformation of spectral levels into discharge rate and indicates the frequency range over which these transformations occur (i.e., spectral selectivity). But how accurate and valid is this model for GBC and MNTB neurons? The validity of the spectral weight model and the RSS technique in general was tested by using the fitted weights and Equation (1) to make predictions of rate responses to arbitrary RSS (and DTF-filtered stimuli, see below) stimuli that were not used for the fitting. Model quality was quantified by the fraction of explained variance, *fv* [see Equation (4), Methods]. *fv* values vary from a maximum of 1 (perfect fit) and decrease with poorer predictions; *fv* can take values <0 when the fit is particularly poor.

As an example of this procedure, Figures 3A3,B3 show the empirical responses to the 64 RSS stimuli comprising the prediction set, plus the predicted rates for the first-order model (*R*_0_ and first order weights, red) and the full-order model that also includes the second order terms, e.g., Equation (1a). For this neuron as well as all others, addition of the second order terms (e.g., Figures 3A3,B3) improved the quality of the fit as assessed by the *fv* value. For the 15 dB stimulus level (Figure 3B3), addition of the second order terms improved the prediction quality from *fv* of 0.46 to 0.54. For the −5 dB stimulus level (Figure 3A3), addition of the second order terms improved the prediction more substantially from *fv* of 0.31 to 0.72. The *fv* metric is approximately equal to the coefficient of determination (*R*^2^; Hays, 1988; see also Young and Calhoun, 2005); the calculated *R*^2^ from the data can, in some instances (e.g., poor estimation of the *R*_0_ parameter), be larger than the corresponding *fv* value. Thus, for the example in Figure 3A3 the full model explains *at least* 72% of the variance in the discharge rates. Note that since *fv* = ~ *R*^2^, an *fv* of 0.72 corresponds to *R* of 0.85. *fv* is a stricter test of the model than correlation coefficient because it is sensitive to deviations of predictions from a slope of 1.0 and a y-intercept of 0.0, whereas correlation coefficient (and thus, *R*^2^) is not. Finally, Figures 3A4,B4 show scatter plots of the predicted rates (model rates) as a function of the empirical rates. Note that the data cluster along the line of equality (slope = 1.0) indicating that the spectral weight function model, e.g., Equation (1a) predicts accurately (at least within the unexplained error due to nearly-Poisson response variability) the discharge rate of this MNTB neuron to arbitrary RSS stimuli that were not used to estimate the parameters of the model.

**Figure 4:**
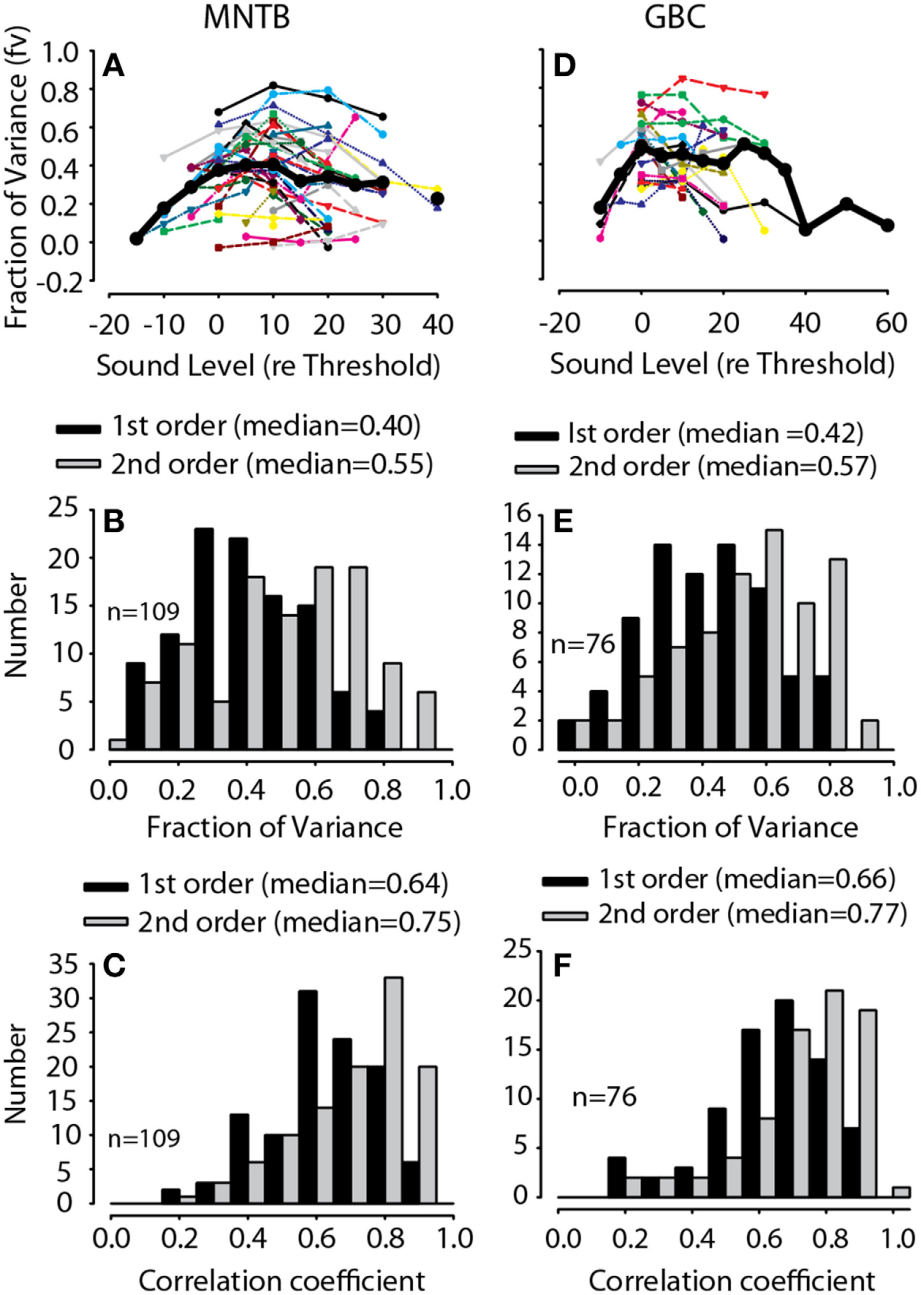
**Predictive validation of the spectral weight function model**. Fraction of variance, *fv*, as a function of RSS stimulus level for MNTB **(A)** and GBC **(D)** neurons; thin lines indicate data for individual neurons and thick black lines the across-neurons mean. Histogram of the maximum fraction of explained variance Equation (4), *fv*, in 42 MNTB neurons (109 total RSS levels) **(B)** and 21 GBC neurons (76 total RSS levels) **(E)** for both the first-order linear model (black) and the full-order model (gray) containing the second-order non-linear terms. The *fv* were computed by predicting the discharge rates to arbitrary RSS stimuli that were not used in the estimation of the spectral weight models. The across-neuron median *fv* for each model is indicated. Histograms of the correlation coefficient, r, relating the predicted spike rate to the empirical rate (e.g., Figure 3B4) are shown in **(C,F)** for MNTB and GBC neurons, respectively.

For all GBC and MNTBs neurons and at all sound levels tested for each neuron, the validity of the spectral weight model was assessed by the accuracy of the rate predictions, as illustrated for the one neuron in Figure 3. Figure 4 plots histograms of the first- and second-order prediction qualities, *fv*, for all MNTB (Figures 4A,B) and GBC (Figures 4D,E) neurons and all sound levels tested. Across the 38 MNTB neurons (*n* = 109 sound levels), the median *fv* was 0.40 [interquartile (IQ) range 0.29–0.54] for the linear first-order model predictions and increased significantly (Wilcoxon signed-ranks test, *Z* = 7.48, *p* < 0.0001) to 0.55 (IQ range 0.37–0.7) for the full-order model. Figure 4D plots the *fv* for the GBC neurons over all sound levels tested. Across the 21 GBC neurons (*n* = 76 sound levels) the median *fv* was 0.42 (IQ range 0.3–0.56) for the linear first-order model predictions and increased significantly (Wilcoxon signed-ranks test, *Z* = 7.57, *p* < 0.0001) to 0.57 (IQ range 0.39–0.68) for the full-order model. In most MNTB and GBC neurons, the linear model alone explained at least 40-90% of the variance (*R* ~ 0.63–0.95).

Although the median *fv* values plotted in Figure 4 may appear low (given the 1.0 maximum value for *fv*), the values underestimate model performance when considering the variance (nearly Poisson) of the empirical discharge rate data. Given just a single repetition and short duration (100 ms) of each RSS stimulus used here, the large response variance will ensure that the *fv* will *always* be less than 1.0; that is, a sizeable fraction of the responses are random and thus not predictable at all by the model. This can be appreciated somewhat by observing the vertical scatter of the responses in Figures 3A4,B4. The error in *fv* due to random effects obscures the actual capabilities of the spectral weighting model, and thus the functional implications of the model for neural processing. There are a variety of ways of correcting for the effects of finite data sampling on such predictions (see David and Gallant, 2005). Here we used the technique described in Young and Calhoun (2005, p. 4448) to correct the *fv* values for such unexplained variance (assuming Poisson variability). After correction, the maximum *fv* we could theoretically obtain with our empirical data was in the range of ~0.5–0.7, which is comparable to that computed by Young and Calhoun (2005) for auditory nerve fibers stimulated with similar RSS stimulus sets. For the examples in Figures 3A4,B4, the estimated maximum *fv* values using the correction method were 0.746 and 0.521, respectively, which were comparable to the empirical *fv* values for the full-order model predictions of 0.73 and 0.47, respectively. In other words, assuming the variance correction above, across the population of neurons (Figures 4B,E) the linear model can generally explain most of the variance in the rate responses of MNTB and GBC neurons to arbitrary RSS stimuli. That the second-order terms only marginally, but still significantly, increased the prediction accuracy suggests that a simple linear weighting is a good model for how MNTB and GBC neurons encode sound spectra. Similar results are reported below for predictions of rate responses to noise stimuli filtered through DTFs.

As another objective measure of model performance that is less susceptible to random errors in rate predictions, Figures 4C,F plot histograms of the first- and second-order prediction qualities, *correlation coefficient* (*r*), for all MNTB and GBC neurons, respectively, and all sound levels tested. Across the 38 MNTB neurons (*n* = 109 sound levels), the median *r* was 0.64 (IQ range 0.54–0.75) for the linear first-order model predictions and increased significantly (Wilcoxon signed-ranks test, *Z* = 8.23, *p* < 0.0001) to 0.75 (IQ range 0.67–0.84) for the full-order model. Across the 21 GBC neurons (*n* = 76 sound levels), the median *r* was 0.66 (IQ range 0.56–0.76) for the linear first-order model predictions and increased significantly (Wilcoxon signed-ranks test, *Z* = 7.55, *p* = 0.003) to 0.77 (IQ range 0.66–0.85) for the full-order model.

MNTB neurons receive a single large calyceal input from the GBCs. To test the hypothesis that MNTB inherit their spectral coding capabilities from GBCs we compared the distributions of *fv* and correlation coefficients plotted in Figure 4. There were no significant differences in *fv* (i.e., Figures 4B,E) between MNTB and GBC neurons for either the first order (Mann-Whitney *U* = 4412, *p* = 0.45) or full order models (Mann-Whitney *U* = 4375, *p* = 0.52). Similarly, there were no significant differences between correlation coefficients (e.g., Figures 4C,F) between MNTB and GBC neurons for either the first order (Mann-Whitney *U* = 4439, *p* = 0.41) or full order models (Mann-Whitney *U* = 4367, *p* = 0.53). These results support the hypothesis that the spectral coding capabilities of GBCs are largely recapitulated in MNTB.

### Functional properties of first order spectral weight functions and their dependence on stimulus level

**Figure 5:**
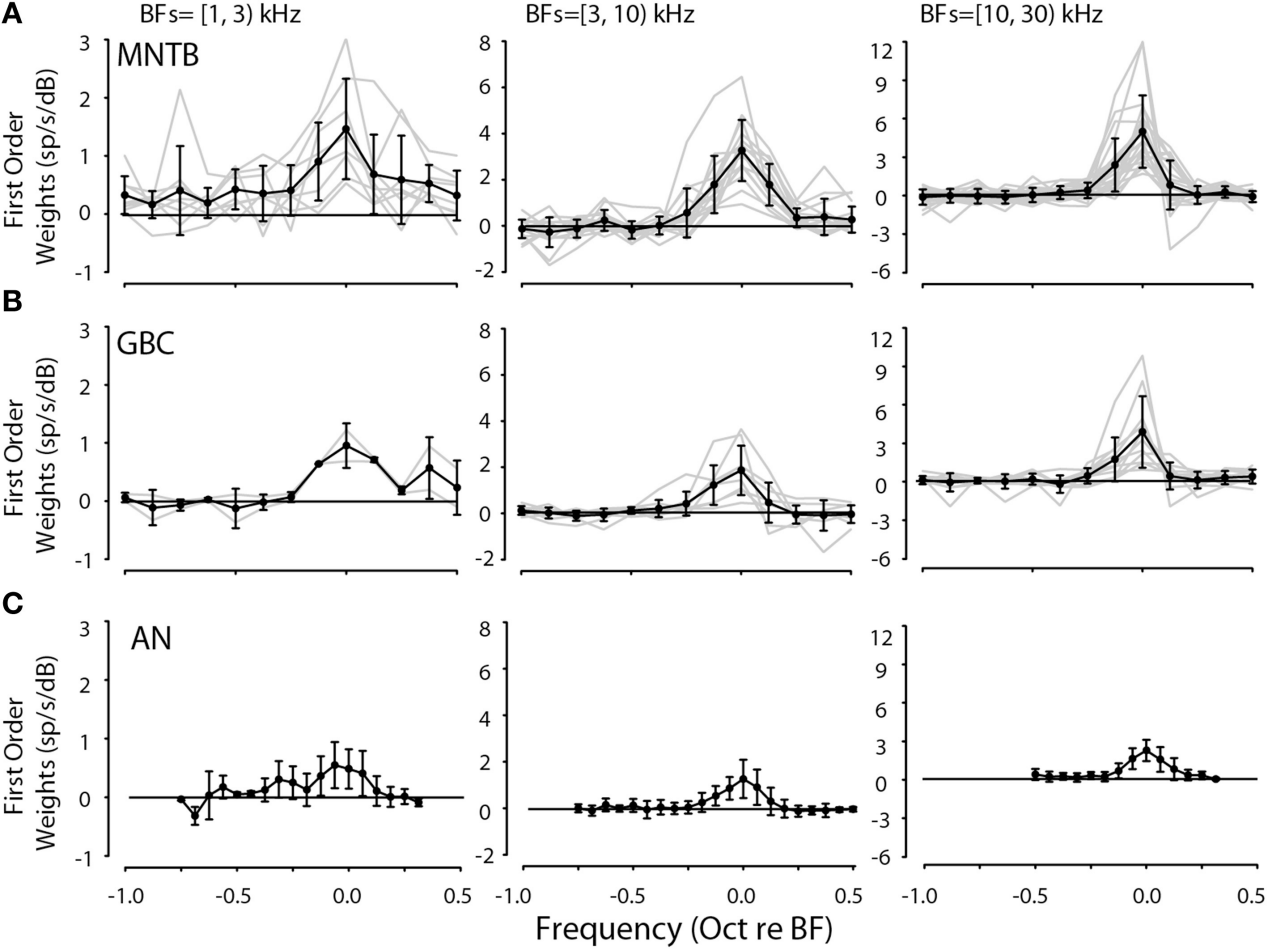
**First-order linear weight functions for all 42 MNTB (A) and 21 GBC (B) neurons studied binned according to BF (BF ranges in upper left of each panel)**. Weight functions were taken from spectral levels in the range of 5–15 dB. Weight function for auditory nerve fibers (AN, **C**) are replotted from Young and Calhoun (2005). In each panel, the light gray lines indicate weight functions for individual neurons while the solid line with symbols and error bars indicate the across-neuron mean weight function ±1 standard deviation. Generally, the weight functions in each range of BFs were similar in terms of shape and magnitude.

The validation of the spectral weight model via accurate predictions to arbitrary stimuli suggests that the properties of the weight functions may have functional meaning (although see Materials and Methods for caveats). The first order (linear) weight functions were examined in one way by averaging weights across all neurons after grouping them into three ranges of BFs: 1–3, 3–10, and 10–30 kHz. Figure 5 shows that the weight functions were quite similar across all neurons in the respective BF groupings when computed at reference sound levels 5–15 dB above threshold. The weight functions in Figure 5 were aligned on the peak of the weight functions (i.e., BF). The gray lines are for individual neurons and the across-neuron average is shown with black lines. The lower frequency BF neurons generally had lower overall weights and the significant weights spanned a larger range of frequencies than those for the mid- to high-BF neurons.

Because the general shapes of the first-order spectral weight functions were quite similar across both MNTB and GBC neurons computed over similar BF ranges, shown in Figures 5A,B, respectively, the functions could then be adequately summarized across neurons and nuclei by two main parameters: maximum weight at BF and the bandwidth of the weight function at half the maximal weight. The bandwidth at half-height (see **Figure 7C**) gives an estimate of the frequency selectivity of the neuron and the maximum weight at BF indicates the strength by which the neuron transforms spectral levels into discharge rate. Across-neuron average first-order spectral weight functions from auditory nerve fibers from the study of Young and Calhoun (2005) are also plotted in Figure 5C for comparison; note that the plotted weights for the auditory nerve fibers are smaller (by approximately a factor of 2) than those for the MNTB and GBC neurons because weights were estimated for twice as many frequency bins (i.e., 1/16 bins/octave) as the weights for the MNTB and GBC neurons (1/8 bins/oct).

**Figure 6:**
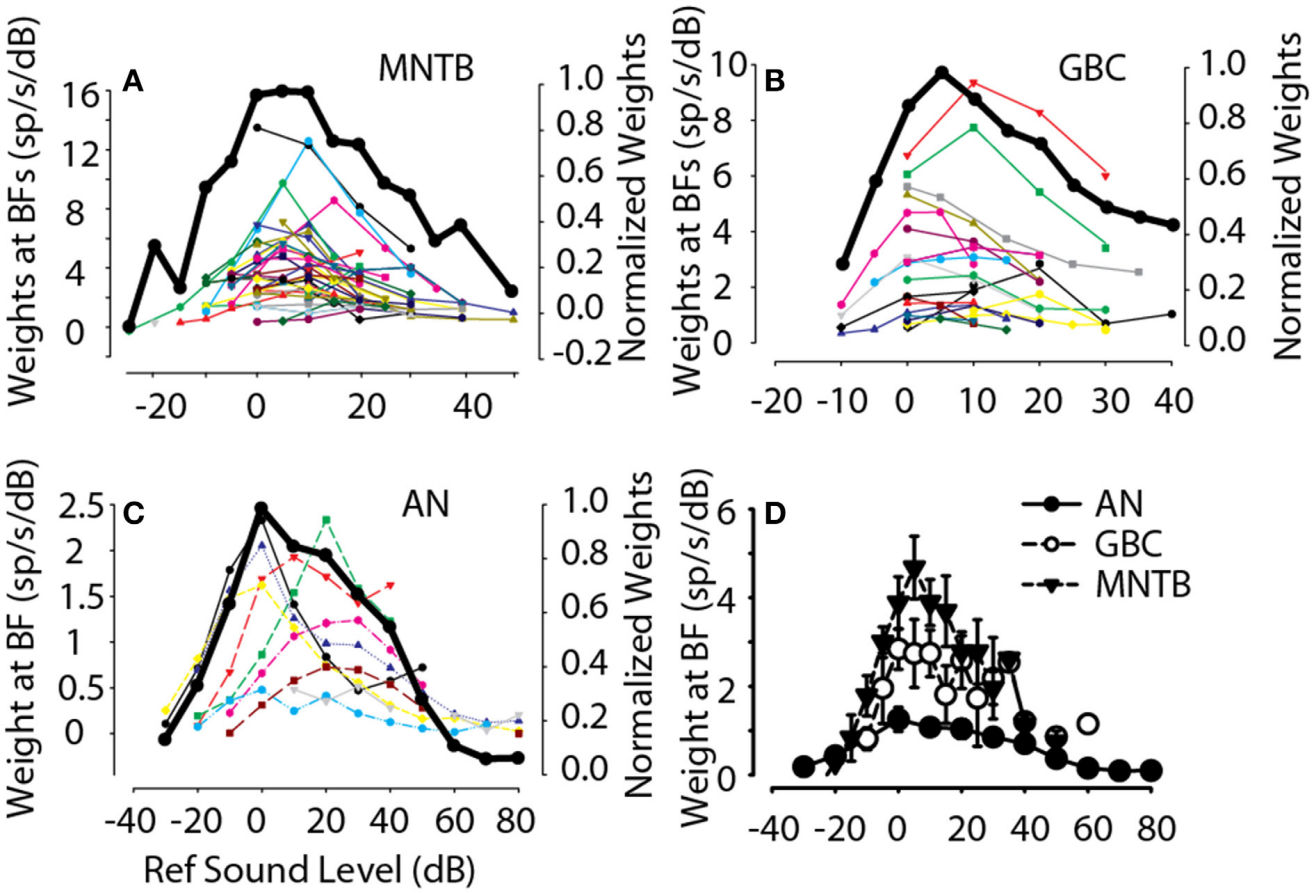
**Dependence of first-order weights at BF on overall sound level**. For all MNTB **(A)**, GBC **(B)**, and ANF **(C)** neurons studied (individual lines and symbols), the first-order weight at BF (left-hand ordinate) is plotted as a function of sound level (re: threshold for flat-spectrum stimulus, Figure 1A). In general, for each neuron the first-order weights were small for low and high sound levels and peaked for moderate sound levels. To summarize this trend, the solid black line shows the across-neuron mean first-order weight as a function of sound level; data for individual neurons was first normalized by the maximum weight before averaging across neurons. **(D)** Summarizes the across-neuron first-order weights at BF in AN, GBC, and MNTB neurons.

As an example of how the spectral weights depended on overall sound level, for each MNTB (Figure 6A), GBC (Figure 6B) and auditory nerve (Figure 6C, from Young and Calhoun, 2005) neuron the maximum spectral weight was plotted as a function of the sound level at which it was measured. To summarize across neurons, each of the functions for the individual neurons was normalized to its maximum weight and then averaged across neurons (Figures 6A–C, black lines, right ordinates). Based on the across-neuron normalized average, the spectral weights of auditory nerve, GBC and MNTB neurons at BF increased with reference sound level, saturated at ~10–15 dB above threshold sound level (i.e., 0 dB), and then decreased with additional increases in level beyond this. These data indicate that across this population of neurons comprising the inhibitory pathway to the LSO, their discharge rates can be modulated in response to broadband stimuli over at least a 60–70 dB range of stimulus levels (−20 to 50 dB re: threshold). While the general level dependence of the maximum spectral weight was similar from auditory nerve to MNTB, the absolute value of the weights were not. Figure 6D plots the across neuron mean spectral weight at BF for the population of auditory nerve fibers (Young and Calhoun, 2005), GBC and MNTB neurons. In general, GBC and MNTB weights exhibited both similar dependencies on RSS sound level and their absolute magnitudes were virtually the same. Both GBC and MNTB weights, however, were substantially larger by a factor of 1.5-2 (even after accounting for the differences in the weights for auditory nerve fibers discussed in the prior paragraph). That is, for every 1 dB increase in stimulation at BF, MNTB and GBCs produce nearly twice as many additional spikes as auditory nerve fibers.

**Figure 7:**
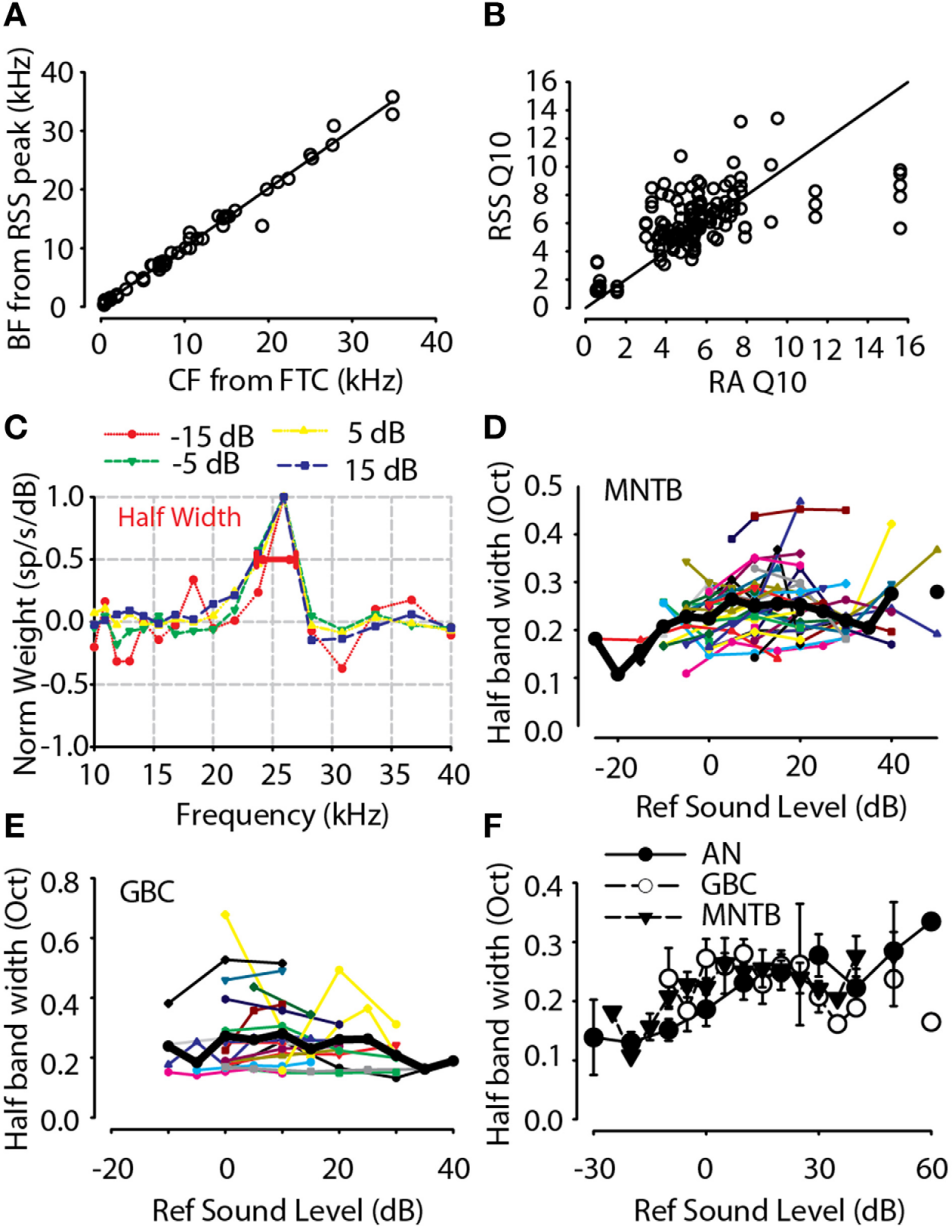
**(A)** BF estimated from the RSS weight function as a function of the CF measured from pure tone frequency-intensity response area. **(B)** Frequency tuning estimated from the RSS weight functions (RSS Q_10_, see text for equation) for all neurons and all levels tested as a function of Q_10_ measured from pure tone frequency-intensity response area (RA Q_10_). **(C)** First-order weight function for one neuron (*BF* = 25.9 kHz) computed over a range of sound levels (−15 to 15 dB). The weight function for each sound level has been normalized by the maximum weight at BF at each particular sound level. For higher sound levels (> −15 dB), the normalized weight function shapes were similar. The bandwidth of the weight functions was computed at one-half the maximum weight at BF (see bar at normalized weight of 0.5). Significant off-BF inhibition (negative weights) can be seen at higher levels (error bars not shown for clarity). **(D)** Half-height bandwidth for 38 high-BF (>3 kHz) MNTB neurons as a function of sound level re: threshold. Lines and points show data for individual neurons while the solid black line shows the across-neuron mean. **(E)** Same as in **(D)**, but for GBC neurons. **(F)** Across-neuron mean spectral selectivity of GBC and MNTB neurons was quite consistent, or level-tolerant, across a wide range levels while the selectivity of AN neurons tended to widen. AN data replotted from Young and Calhoun (2005).

In addition to characterizing the spectral weights at BF, the BF estimated from the weight function for MNTB neurons was positively correlated with the CF estimated from pure tones (i.e., the frequency at which a neuron just responds at the lowest sound level) and shown in Figure 7A; the linear regression of the data indicated a strong correlation (*R* = 0.992, *p* < 0.0001) with a relationship of the form *BF*_*RSS*_ = 1.0 × *CF*_*tones*_ + 0.18. Thus, as an additional validation of the RSS technique, the properties of the spectral weight functions can yield accurate measurements of traditional metrics for frequency selectivity, BF and Q_10_. Bandwidths as computed above can be converted to *Q*_10_ using the approximation of Young and Calhoun (2005) *Q*_10_ = 1/[ln(2) × *octave halfwidth*], where *octave halfwidth* was computed from the formulae earlier in this section. Figure 7B shows the Q_10_ estimated from the first-order weight functions for MNTB neurons compared with Q_10_ measured with tones. The linear regression of the Q_10_estimated from the first-order spectral weight functions (for all neurons measured at each stimulus level tested) as a function of the empirical Q_10_ measured with pure tones was significant (*R* = 0.85, *P* < 0.0001, *n* = 144) with a relationship of the form *Q*_10 *RSS*_ = 0.9 × *Q*_10 *tones*_ + 1.0.

### Level tolerance of spectral coding

In addition to the spectral weight dependence on stimulus level, Figures 7C,D shows how the bandwidth of spectral tuning depended on stimulus level for GBC and MNTB neurons. First, Figure 7C shows an example of how the bandwidth of the weighting function at half the maximum weight was calculated for one MNTB neuron. Generally for stimulus levels above 0 dB, the weights at BF were positive and followed the general trends with overall stimulus level as that shown in Figure 6. The neuron in Figure 7C also showed significant off-BF inhibition/suppression (i.e., negative spectral weights) on the high-frequency side for high stimulus levels. By plotting normalized spectral weight functions (i.e., each weight function normalized by its maximum weight), the neuron in Figure 7C also revealed that the bandwidth was not affected much by changing the sound level over a range of 40 dB, 30 dB of which are shown (−15 to 15 dB re: threshold). Following Young and Calhoun (2005), for each neuron and for each sound level tested, the bandwidth corresponding to half the maximal spectral weight was computed on an octave scale using the formula *log*(*F*_*upper*_/*F*_*lower*_)/*log*(*2*). Figures 7D,E show the general stimulus level independence of the half-maximum weight bandwidth for all high-BF (>3 kHz) MNTB and GBC neurons, respectively. As stimulus levels increased, the spectral selectivity remained generally constant. The across-neuron mean bandwidths (Figures 7D,E, thick black lines) were constant in the range of ~0.2–0.225 octaves over at least a ~60 dB range of sound levels for both GBC and MNTB neurons. The observed bandwidths for four low-BF MNTB neurons (BF < 2 kHz, not shown in Figure 7D) were quite broad relative to the higher-BF neurons, with widths in the range of 1.0–1.2 octaves.

Figure 7F shows the across neuron mean bandwidths for auditory nerve fibers (Young and Calhoun, 2005), GBC and MNTB neurons. While the auditory nerve fiber bandwidths generally increased with increasing sound level (see also Young and Calhoun, 2005), the bandwidths for GBC and MNTB neurons remained quite constant, and similar, suggesting that the spectral selectivity of the inhibitory pathway through GBC and MNTB to LSO is invariant to sound level. Over a range of 50 dB (from −10 to 40 dB re: threshold) the high BF (>2 kHz) GBC and MNTB neurons had median bandwidths of 0.21 (IQ range = 0.17–0.27) and 0.23 (IQ range = 0.19–0.26), respectively, which were not significantly different than each other (Mann-Whitney *U* = 3572, *p* = 0.34). These data are consistent with the hypothesis that MNTB neurons inherit most of their level tolerance for spectral coding from the GBCs.

### Prediction of rate responses for DTF-filtered broadband noise stimuli

A primary function of the neural circuit comprising the LSO is the computation of the ILD cues for sound localization (Tollin, 2003). The inhibitory pathway to the LSO including the auditory nerve, GBC and MNTB should thus accurately encode sound spectra. Therefore, as an additional test of the biological relevance and also generalizability of the spectral weight model, in 14 MNTB neurons, we also collected responses to 100-ms duration noise that was filtered by the DTFs measured in each animal prior to the physiological experiments (see Tollin and Koka, 2009a,b for methods). The DTFs covered 627 locations in the frontal hemisphere, which contain spectral notch cues. These DTF-filtered stimuli provide a more ecologically-relevant set of test stimuli because they contain the spectral components necessary for sound localization based on ILDs. Responses to DTF stimuli were predicted for each neuron using the spectral weighting functions estimated with the RSS stimuli. Compared to the RSS spectra (e.g., Figure 1), the DTF spectra are much smoother, except perhaps at frequencies corresponding to the spectral notches; an example DTF spectrum is shown in **Figure 11A**. In order predict the responses using the spectral weight functions, first the energy in the DTF spectra was resampled into frequency bins corresponding exactly to those bins (8 bins/oct) used in the RSS stimulus set. These DTF spectra were also corrected for speaker calibration (either directly via the *in situ* speaker calibration filter, or *post-hoc*) and then reexpressed in terms of dB level relative to the reference stimulus level. Both the DTF and RSS stimuli were presented at different reference sound levels in steps of 5 or 10 dB. For each DTF stimuli set, the spectral weight function model used for prediction was taken from the RSS data set that was measured with a reference level nearest (within 5–10 dB) the mean DTF sound level computed at the neuron BF. This method is adapted from that used by Young and Calhoun (2005).

**Figure 8:**
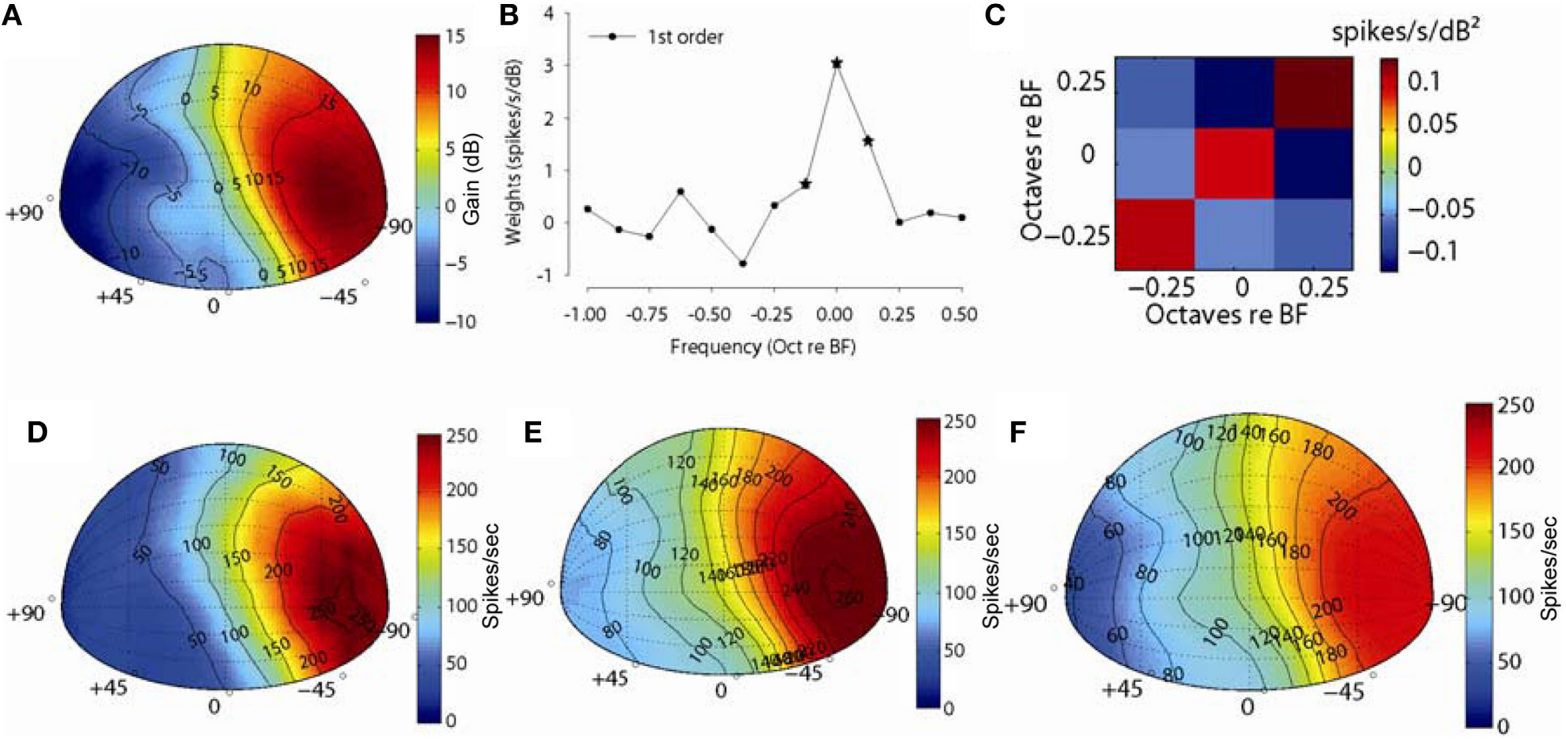
**Discharge rate predictions to broadband noise filtered through acoustical head related directional transfer functions (DTFs). (A)** Spatial plot of the spectral levels (dB) for DTF filtered broadband noise stimuli corresponding to the CF of one neuron (10 kHz). **(B,C)** show the first- and second-order weights, respectively, used for predicting rate responses to DTF stimuli. **(D)** Spatial plot of empirical discharge rates for the 325 (out of 627) front-hemisphere DTF stimuli and predicted rates with first-order **(E)** and full-order **(F)** spectral weight models.

Figure 8 shows an example for one MNTB neuron (*CF* = 10 kHz). Figure 8A shows the spatial plot of the acoustical gain of the head and pinnae of the DTF at a frequency bin corresponding to the CF (10 kHz) of the neuron (positive gain indicates amplification by the head and pinnae, negative gain indicates attenuation). The acoustic gain was maximal (~15 dB) for sounds ipsilateral to the ear being measured, and the gain was reduced systematically for sound source locations away from this point (see Tollin and Koka, 2009a for detailed analyses of acoustical gains of head and pinnae in the cat). Figure 8B shows the first order weight function at sound level 20 dB above threshold and Figure 8C shows the second order terms. The first order terms with star symbol are the weights which optimized the *fv* (see Methods). The spatial distribution of empirical responses to DTF-filtered stimuli presented at 20 dB re: threshold and the first and full-order model predictions are shown graphically in Figures 8D–F. The distribution of empirical responses (Figure 8D) roughly matches the distribution of acoustical gain corresponding to the neuron BF, as might be expected. The spatial distributions of responses predicted by the linear- (Figure 8E) and full-order (Figure 8F) spectral weight models are similar to the empirical rate distributions (Figure 8D) in terms of the areas of space over which the neuron responded. The *fv* values for prediction for this neuron were 0.62 and 0.71 and spatial correlation coefficients were 0.98 and 0.99 for the first-order and all order models, respectively. In general, even the first-order linear model produces predictions of the spatial receptive field structure nearly perfectly.

**Figure 9:**
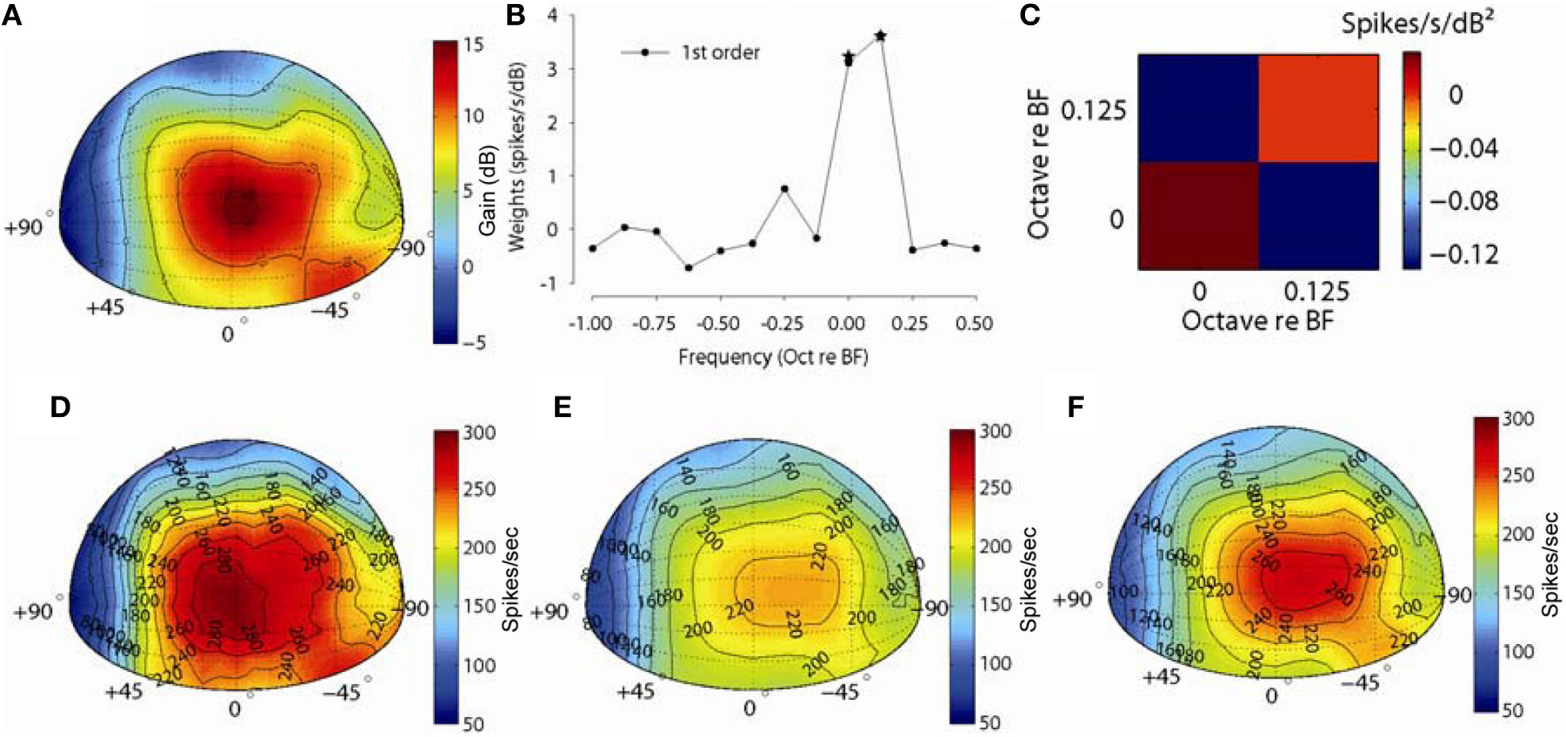
**Discharge rate predictions to broadband noise filtered through acoustical head related directional transfer functions (DTFs)**. Same as in Figure 8 but for a MNTB neuron with a CF of 15.4 kHz.

Figure 9 shows an example for another MNTB neuron (*CF* = 15.4 kHz). Figure 9A shows the spatial plot of the acoustical gain of the head and pinnae of the DTF at a frequency bin corresponding to the CF (15.4 kHz) of the neuron (positive gain indicates amplification by the head and pinnae, negative gain indicates attenuation). The acoustic gain was maximal (~15 dB) for sounds immediately in front, and the gain was reduced systematically for sound source locations away from this point. As such, the spatial distribution of acoustical gain is somewhat more complex than that in the example in Figure 8. Figure 9B shows the first order weight function at sound level 15 dB above threshold and 9C shows the second order terms. The first order terms with star symbol are the weights which optimized the *fv*. The spatial distribution of empirical responses to DTF-filtered stimuli presented at 15 dB re: threshold and the first and full-order model predictions are shown graphically in Figures 9D–F. The distribution of empirical responses (Figure 9D) roughly matches the distribution of acoustical gain corresponding to the neuron BF, as might be expected. The spatial distributions of responses predicted by the linear- (Figure 9E) and full-order (Figure 9F) spectral weight models are similar to the empirical rate distributions (Figure 9D) in terms of the areas of space over which the neuron responded. The *fv* values for prediction for this neuron were 0.72 and 0.78 and the spatial correlation coefficients were 0.96 and 0.97 for the first-order and all order models, respectively. As with the example neuron shown in Figure 8, a linear weighting of the stimulus spectrum provided by the DTFs producted predictions of the structure of the neuron's spatial receptive field nearly perfectly.

**Figure 10:**
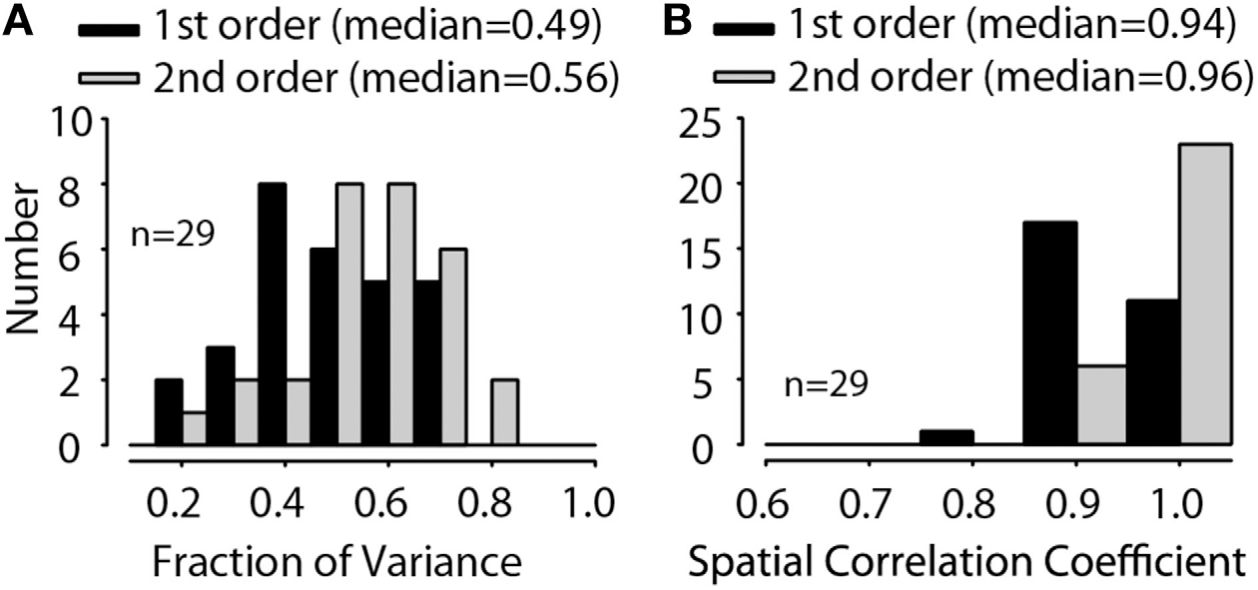
**Distributions of the fraction of variance, *fv* (A), and the spatial correlation coefficient (B) for 14 MNTB neurons (tested at 29 overall stimulus levels) for both the first-order linear model (black) and the full-order model (gray)**. The across-neuron median *fv* and spatial correlation coefficient for each model is indicated.

To summarize the predictions, Figure 10A shows the histogram of the quality of fit *fv* values for the 14 MNTB neurons studied at different sound levels (*n* = 29 levels). The *fv* values from the full-order weight function model (median = 0.56, IQ range 0.47–0.67) were significantly (Wilcoxon signed-ranks test, *Z* = 4.7, *p* < 0.0001) different from the *fv* values (median = 0.49, IQ range 0.39–0.61) from first-order model. Over all, the *fv* values from the prediction of these DTF-filtered stimuli accounted for ~60% of the variance in the empirical data, and they were generally better than the *fv* values for RSS data sets. Given just a single repetition and short duration (100 ms) of each DTF-filtered stimulus used here, the large response variance will ensure that the *fv* will always be less than 1.0; that is, some fraction of the responses are random and thus not predictable at all by the model. When *fv* is corrected (as described above) for this, the linear spectral weight model can account for virtually all of the variance in the empirical rates in response to DTF-filtered stimuli. Figure 10B shows the histogram of spatial correlation coefficients for the quality of predictions. The spatial correlation coefficient shows how well the models can predict the actual shape of the acoustics via discharge rate. The spatial correlation coefficients values from the full-order weight function model (median = 0.97, IQ range 0.95–0.98) were significantly (Wilcoxon signed-ranks test, *Z* = 4.62, *p* < 0.0001) different from the *fv* values (median = 0.94, IQ range 0.93–0.96) from first-order model.

### The spectral modulation selectivity of MNTB neurons is sufficient to encode the modulation spectra of the cues to sound location

The MNTB is hypothesized to play an essential role in the encoding of the ILD cues to sound location as it provides the direct inhibitory input necessary for the computation of ILD by LSO (Tollin and Yin, 2002a,b; Tollin, 2003). Acoustically, ILDs are defined as the binaural difference in the sound spectra at the two ears. Examination of the ILD cues to location in a variety of mammalian species (Koka et al., 2008, 2011; Greene et al., 2014), including the cat (Tollin and Koka, 2009b) have demonstrated that large ILDs are produced in part due to the spectral notch cues induced by diffraction of sound by the pinnae. The results here have demonstrated that MNTB neurons are capable of accurately encoding via spike rate the monaural spectral information contained in DTF-filtered stimuli (Figures 8–10). This result implies that the spectral modulation resolving capacities of MNTB neurons must encompass the spectral modulations, or spectral-envelope frequency, of the ensemble of DTFs.

**Figure 11:**
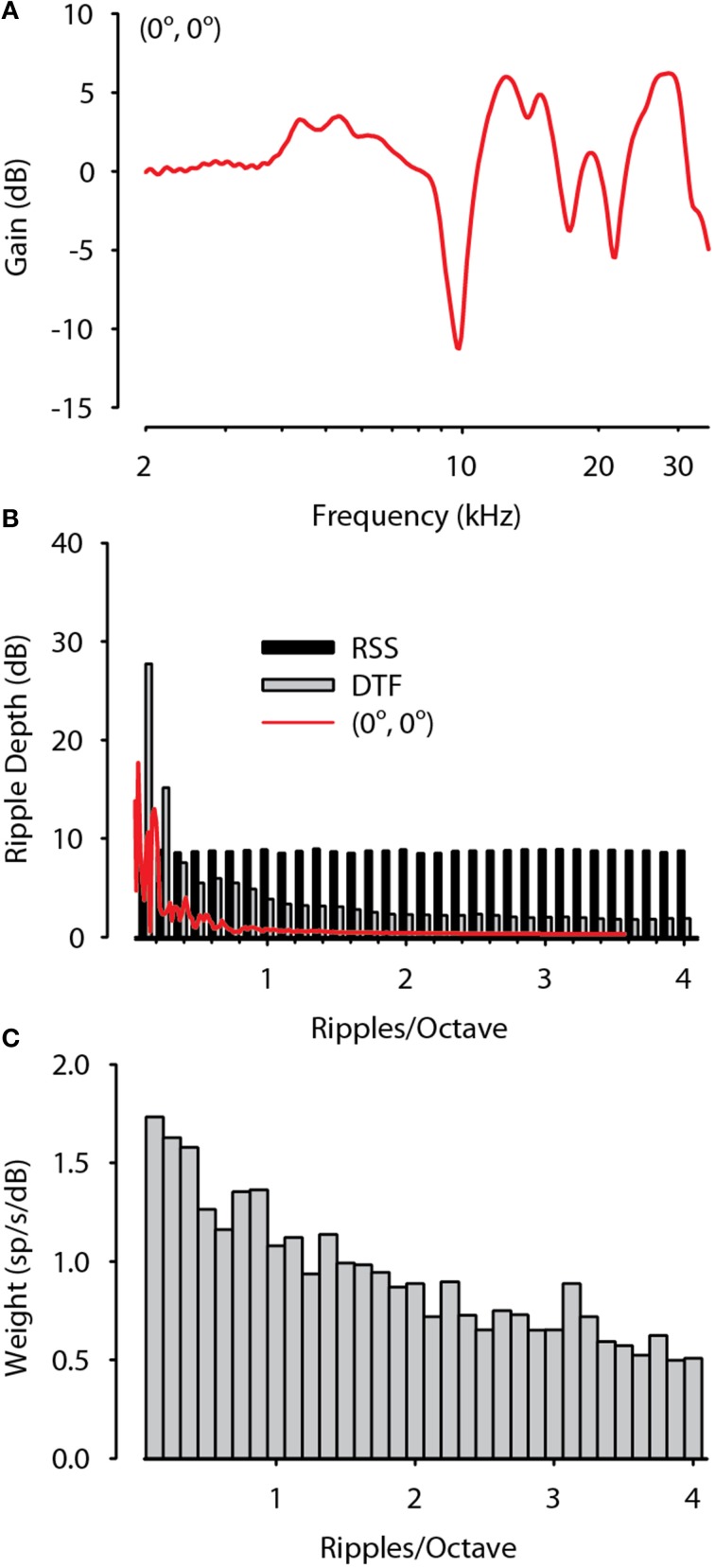
**Spectral modulation selectivity of MNTB neurons are sufficient to encode the spectral modulations contained in spatial head related transfer functions. (A)** The directional transfer function (DTF) for a sound source located at (0°, 0°). **(B)** Spectral modulation distribution of the DTF in **(A)**, the ensemble of RSS stimuli, and the ensemble of DTFs used in this study plotted as mean ripple depth (dB) as a function of ripples/octave. DTFs have large ripple depth at low ripples/octave while RSS have constant mean ripple depth up to at least 4 ripples/octave. **(C)** Spectral modulation selectivity of the population of MNTB neurons in terms of spectral weighting (spikes/s/dB) as a function of ripples/octave. MNTB neurons preferentially resolve low spectral modulations consistent with those provided by the DTFs.

In order to test this hypothesis, following the method described by Macpherson and Middlebrooks (2003, p. 437) we computed the distributions of spectral modulations of the DTFs used for this study as well as the ensemble of RSS stimuli themselves. We then compared these distributions to the spectral modulation selectivity of the MNTB neurons as computed from the spectral weighting functions. Figure 11A shows the DTF for a midline (0°, 0°) sound source and the red line in Figure 11B shows the spectral modulation for this sound in terms of spectral ripple (or spectral envelope) depth (in dB) as a function of spectral ripple frequency (in ripples/octave) for this location. The DTFs of cats for frontal hemisphere locations contain pronounced features such as spectral peaks and deep notches for frequencies above ~8 kHz (Rice et al., 1992; Tollin and Koka, 2009a). The resulting DTF ripple spectrum for the (0°, 0°) source shows two prominent ripples >10 dB over the range of 0.25–1 ripples/octave. Across all source locations in the frontal hemisphere the mean DTF ripple depth was >5 dB for ripples/octave < ~1.5 and was lower for ripples/octave higher than 2. The modulation spectrum of the ensemble of RSS stimuli used in this study was flat up to around 4 ripples/octave, demonstrating that the RSS stimuli were sufficient to probe the spectral modulation coding capability of the neurons at least over this range of modulation.

The RSS spectral weighting functions can be used to estimate the spectral resolving power of neurons based on rate responses. The high spatial correlation coefficients between predicted and empirical spike rates for DTF-filtered stimuli imply that MNTB neurons can accurately encode the spectral envelopes contained in these stimuli. To more directly examine whether MNTB neurons have sufficient spectral resolving power to encode the modulation spectra of directional information contained in the DTFs (Figure 11B) we decomposed the spectral weighting functions for MNTB neurons with BFs > 3 kHz as a function of ripples/octave. The results shown in Figure 11C reveal that MNTB neurons prefer low ripple densities, as indicated by higher spectral weights (spikes/s/dB), indicating that they prefer broad spectral features, similar to those produced by the distributions of DTFs. Thus, the spectral modulation sensitivities of the population of MNTB neurons studied here are sufficient to encode the biologically-relevent spectral modulations contained in the directional information conveyed by the DTFs. This ability is essential in order for MNTB neurons to transmit information about stimulus spectrum via rate to the LSO for accurate encoding of the ILD cues to location.

## Discussion

The classical function of the MNTB is to provide inhibitory input to the LSO necessary for encoding the ILD cue to location (Yin, 2002; Tollin, 2003). However, based largely on *in vitro* studies, MNTB neurons have recently been described and intensely studied in terms of their abilities to encode *temporal* information (Taschenberger and Von Gersdorff, 2000; Trussell, 2002; Schneggenburger and Forsythe, 2006). In addition to the input to ipsilateral LSO, the MNTB sends glycinergic projections to the MSO, the superior paraolivary nucleus, and the ventral nucleus of the lateral lemniscus (Spangler et al., 1985; Banks and Smith, 1992; Sommer et al., 1993; Smith et al., 1998). The projection of MNTB to MSO is considered to be an important mechanism for the encoding of ITDs by the MSO in small mammals (Brand et al., 2002; Pecka et al., 2008). The hypothesized function of these projections is to provide temporally-precise inhibition. Here, however, we reexamined the traditional hypothesis that MNTB neurons provide the inhibitory input to the LSO required for ILD encoding and thus encode the shapes of sound spectra via discharge rate.

### The MNTB as a spectral analyzer

The ability of receptive field models to generate response predictions to arbitrary stimuli is essential to establishing functional properties based on the model parameters (see Methods). The spectral weight models here produced predictions that supported the hypothesis that MNTB neurons and their inputs, the GBCs, encode stationary spectra via discharge rate. Both GBC and MNTB neurons were well modeled by a linear weighting of spectra, consistent with auditory nerve (Young and Calhoun, 2005; Reiss et al., 2007) and other CN neurons (Yu and Young, 2000; Yu, 2003). The non-linear terms reflected the need to model the curvature of the discharge rate-level function near threshold and at high sound levels. Addition of the non-linear terms marginally, but significantly, increased the predictive capacity of the model. Given the predictive validation of the model and because we also accounted for possible non-linear aspects, the properties of the weight functions, like bandwidth, sideband suppression/inhibition (hereafter referred to as inhibition), BF and the magnitudes of the weights have functional meaning. See Christianson et al. (2008) for the importance of non-linearities for predictive validation.

MNTB neurons were able to accurately encode via rate the complex spectral shapes of the DTFs produced by the directional filtering of sounds by the head and pinnae. A comparison of the spectral modulation spectra of cat DTFs (Figure 11B) and the spectral resolution of MNTB neurons as assessed from the RSS-derived spectral weight functions (Figure 11C) revealed that there was sufficient spectral resolution of MNTB neurons to adequately encode the spectral modulations contained in the DTFs of cats. Hence, the MNTB is capable of providing via rate response the full range of spectral information necessary to localize sound based on ILD or spectral shape cues in the frontal hemisphere.

### Level tolerance for spectral coding in the neural circuit for interaural level difference cue computation

The spectral-tuning bandwidths of GBC and MNTB neurons were similar and remarkably stable over a wide range of intensities (Figures 7D,E), or level tolerant. Level tolerance may emerge from or be sustained by on- and/or off-BF inhibition. Spectral coding by auditory nerve fibers has been shown to be not as level tolerant as that seen here and in other areas of the auditory system (Young and Calhoun, 2005; Yu and Young, 2013). We hypothesize that level tolerance may be required for encoding of the spectral levels of stimuli via discharge rate necessary for level-tolerant ILD coding (see Tsai et al., 2010). Level-tolerant frequency selectivity has many hypothesized computational attributes, including the capability to create a more accurate neural representation of spectra (Suga, 1977; Sadagopan and Wang, 2008), computation of ILDs (Tsai et al., 2010) and the use of spectral cues for sound localization over a wide range of sound levels (Tollin et al., 2005, 2013; Gai et al., 2013). In the context of the GBC-MNTB-LSO circuit, level-tolerance may function to preclude confounds between sound level and the bandwidth of neural spectral selectivity. This is necessary because the ILD cue in cats can vary by as much as ±40 dB (Tollin and Koka, 2009b). Thus, the capabilities of individual afferents to LSO, including the GBC and MNTB, to maintain consistent spectral coding over a 40 dB range or more is essential. Similar invariance, but to varying degrees, has been reported in AN (Young and Calhoun, 2005), CN (Yu and Young, 2000), inferior colliculus (Yu and Young, 2013), and auditory cortex (Suga and Tsuzuki, 1985; Ehret and Schreiner, 1997; Sutter, 2000; Barbour and Wang, 2003; Sadagopan and Wang, 2008). Here we demonstrate that spectral coding was relatively more invariant to level in GBC and MNTB neurons than in AN fibers (Figure 7F), which suggests that some degree of invariance is produced at the level of the cochlear nucleus and/or MNTB itself.

One potential mechanism for level tolerance may be on- or off-BF inhibition to GBC and/or MNTB neurons. The BF and frequency selectivity estimated from the weight functions were highly correlated with measures using tones, CF and Q_10_. The broadband and stationary nature of the RSS stimuli also allowed for revelation of properties not easily observable with tones. For example, 49% of high-BF (>3 kHz) MNTB neurons showed significant off-BF inhibition. The lack of observable off-BF effects in other neurons does not preclude on-BF or other inhibitory effects. Because similar forms of inhibition (or suppression) are observed in auditory nerve (Sachs and Kiang, 1968) and GBCs (Caspary et al., 1994; Kopp-Scheinpflug et al., 2002) that provide input to MNTB, it cannot be determined here whether this off-BF inhibition was created and/or enhanced directly at the MNTB.

It is known, however, that MNTB neurons do indeed receive direct inhibitory inputs (Adams and Mugnaini, 1990) from a variety of sources including the ventral nucleus of the trapezoid body (Kuwabara et al., 1991; Albrecht et al., 2014), dorsomedial periolivary nucleus (Kuwabara et al., 1991; Schofield, 1994) and intrinsic collaterals from neighboring MNTB neurons (Bledsoe et al., 1988; Kuwabara and Zook, 1991). These sources are comprised of neurons containing GABA and glycine (Helfert et al., 1989). *In vitro* studies have demonstrated that glycine, and GABA, can influence MNTB responses directly (Banks and Smith, 1992; Wu and Kelly, 1995; Turecek and Trussell, 2001; Awatramani et al., 2004, 2005; Lu et al., 2008). Glycine also acts presynaptically to enhance glutamate release by the calyx onto MNTB neurons (Turecek and Trussell, 2001). A possible function of inhibition has been suggested by *in vivo* studies where sideband inhibition in the frequency-intensity response areas has been demonstrated (Kopp-Scheinpflug et al., 2003; Tolnai et al., 2008a,b). Kopp-Scheinpflug et al. (2008) reported 10/19 (53%) neurons had off-BF inhibition, comparable to the 49% observed here. Tolnai et al. (2008a,b) also reported some high-CF MNTB neurons with off-BF inhibition. Tsuchitani (1997) did not report any suppression of spontaneous activity by off-BF tones in 40 MNTB neurons in cat.

By independently analyzing the acoustically-evoked pre-potentials and action potentials of the complex waveforms (e.g., Figure 2A), Kopp-Scheinpflug et al. (2003) suggested that off-BF inhibition was enhanced by some mechanism acting at MNTB neurons directly (although see McLaughlin et al., 2008). Kopp-Scheinpflug et al. (2008) subjected MNTB neurons to acoustic stimulation before, during, and after iontophoretic application of the glycine receptor antagonist strychnine. Strychnine was found to enhance or reduce discharge rates. Rate reductions were most common for spontaneous activity and for sound-evoked responses throughout the excitatory response areas, with the largest reductions occurring for frequencies near CF. Outside the excitatory area, strychnine often caused rate increases, consistent with the hypothesis that blockage of glycine action reduced the strength of putative on/off-BF inhibition. Frequency selectivity in these latter neurons decreased (i.e., poorer selectivity). Comparable results have been observed in CN (Caspary et al., 1994; Kopp-Scheinpflug et al., 2002). Thus, one function of direct glycinergic inhibition to MNTB may be to increase frequency selectivity. There is evidence from prior studies that MNTB neurons may be more sharply tuned than the GBCs that provide the input (Kopp-Scheinpflug et al., 2003) and the ipsilateral excitatory responses of LSO neurons to which MNTB projects (Tsuchitani, 1997). Consistent with these findings our results here also revealed a slight, but significant, increase in frequency selectivity (Figure 2C) in MNTB neurons over GBCs when assessed with tones; however, there was no difference in frequency selectivity between MNTB and GBC when assessed with the broadband RSS-derived spectral weight functions (Figure 7F). The later result, along with the striking similarities between GBCs and MNTB neurons in terms of the characteristics of the spectral weight functions, and the similar rates of spontaneous activity are consistent with the hypothesis that MNTB neurons largely inherit their spectral coding capabilities from GBCs via the calyx of Held.

Given the anatomical and *in vitro* evidence for inhibitory inputs to MNTB, it was surprising that we and others find only a portion of neurons exhibiting off-BF inhibition. The spectral coding properties of MNTB and their GBC inputs were found here to be remarkably similar, consistent with the large calyceal input. Perhaps inhibition is on-BF and thus matched to the excitatory RF. Such mechanisms have been demonstrated in whole-cell studies by Wehr and Zador (2003) in auditory cortex and Gittleman et al. (2009) in inferior colliculus. Alternatively, there may be temporal interactions of excitation and inhibition that cannot be revealed by extracellular recordings (e.g., Xie et al., 2007). Perhaps the spectrally stationary stimuli used here are not sufficient to evoke underlying inhibitory mechanisms in some neurons.

### A solution to the temporal “correspondence problem” for ILD computation—the MNTB encodes sound spectra with high temporal precision

As mentioned in the Introduction, the MNTB and bushy cells have recently been mostly described, and studied, in regards to their exquisite abilities to encode *temporal* aspects of sounds, not spectral. Indeed the inhibitory inputs to MNTB may play important roles in temporal processing that would not be revealed in the present study. It has been suggested that enhanced temporal precision allows transient or complex stimuli with slowly varying envelopes to be more precisely encoded in MNTB than their afferents (Joris and Yin, 1998; Kopp-Scheinpflug et al., 2003, 2008; Tollin and Yin, 2005). Inhibition may thus help to extract and represent spectrotemporally modulated envelopes of natural sounds, like speech, animal vocalizations, or transients like rustling leaves and snapping twigs. To compute the ILD cue to location in these kinds of signals at the LSO requires care (see Tollin, 2003). As suggested by Joris and Yin (1998) and Tollin (2003), there is a time correspondence problem for ILD computation not unlike the classical spatial correspondence problem for stereoscopic vision (Julesz, 1971). The anatomical and biophysical specializations in the GBC-MNTB-LSO circuit may minimize the relative timing delays and the jitter in synaptic delays so that ILD can be computed at corresponding points in frequency and *time* (Joris and Yin, 1998; Tollin, 2003).

The precise spectrotemporal encoding of sound by LSO afferents is well-matched by correspondingly short integration times of LSO neurons. Contralateral stimulus-evoked IPSPs recorded in LSO neurons have relatively long durations (3.2–8.1 ms), nearly twice the duration of ipsilaterally-evoked EPSPs (1.5–4.2 ms) (Sanes, 1990). Functionally, however, contralateral inhibition suppresses ipsilaterally-evoked discharges of LSO neurons for only ~1.0–2.0 ms (Sanes, 1990; Wu and Kelly, 1992; Joris and Yin, 1995; Park et al., 1996; Irvine et al., 2001). The integration time for the comparison of afferent inputs by LSO neurons is sufficiently short so that the ILD is only computed over brief, temporally corresponding portions of the sounds at each ear, thus solving the temporal correspondence problem for ILD computation. Integration times of 1–2 ms would presumably allow ongoing ILD computation for envelope fluctuations up to ~1 kHz before synaptic inputs would be temporally integrated. Some LSO neurons can track ILDs in binaural amplitude-modulated stimuli at rates up to 800 Hz (Joris and Yin, 1995). Such short integration times in the MNTB-LSO spectral processing pathway are wholly consistent with spectral integration times of 1–5 ms estimated from human and animal psychophysical sound localization studies (Hofman and van Opstal, 1998; Tollin and Henning, 1999; Martin and McAnally, 2008; Gai et al., 2013). In order to reconcile the exquisite temporal and spectral coding capabilities of MNTB and their GBC inputs, we propose that together with the anatomical and biophysical specializations for timing in the GBC-MNTB-LSO complex, the linear spectral coding mechanisms demonstrated here may ultimately function synergistically to allow ILDs to be computed for biologically-relevant complex stimuli with rapid spectrotemporally-modulated envelopes such as speech and animal vocalizations and moving sound sources.

### Conflict of interest statement

The authors declare that the research was conducted in the absence of any commercial or financial relationships that could be construed as a potential conflict of interest.
